# Structural insights into distinct filamentation states reveal a regulatory mechanism for bacterial STING activation

**DOI:** 10.1128/mbio.00388-25

**Published:** 2025-08-14

**Authors:** Yuchao Yang, Yueyue Liu, Xue Ma, Xuan Zhao, Jian Cao, Yu Liu, Shanqin Li, Jing Wu, Yuanzhu Gao, Lianwan Chen, Changxin Wu, Guijun Shang, Sheng Liu, Defen Lu

**Affiliations:** 1 Shanxi Key Laboratory for Modernization of Traditional Chinese Veterinary Medicine (TCVM), College of Veterinary Medicine, Shanxi Agricultural University, Jinzhong, China; 2 College of Life Sciences, Shanxi Agricultural University 74600, Taiyuan, China; 3 Institute of Pediatrics, Shenzhen Children’s Hospital, Shenzhen, China; 4 Department of Infectious Diseases, Shenzhen Children’s Hospital, Shenzhen, China; 5 Cryo-EM Center, Southern University of Science and Technology, Shenzhen, China; 6 Key Laboratory of Biomacromolecules (Chinese Academy of Sciences), National Laboratory of Biomacromolecules, Institute of Biophysics, Chinese Academy of Sciences, Beijing, China; 7 The Key Laboratory of Medical Molecular Cell Biology of Shanxi Province, Institutes of Biomedical Sciences, Shanxi University, Taiyuan, China; 8 School of Pharmacy, East China Normal University 12655, Shanghai, China; Case Western Reserve University School of Medicine, Cleveland, Ohio, USA

**Keywords:** TIR-STING, 3′3′-c-di-GMP, filament, CBASS, CD-NTases, Cap proteins

## Abstract

The cyclic oligonucleotide-based antiphage signaling system (CBASS) is a bacterial immune mechanism that was evolutionarily linked to the eukaryotic cGAS–STING pathway, which protects against phage infection through abortive cell death. CBASS operons encode cyclic dinucleotide synthases (CD-NTases) and effector proteins (Caps), such as bacterial STING, which senses cyclic dinucleotides like 3′3′-c-di-GMP to trigger defense. Although bacterial STING oligomerizes into filaments upon ligand binding, the functional roles of distinct filament states remain unclear. Here, we resolve cryo-EM structures of *Epilithonimonas lactis* TIR-STING (*El*STING) bound to 3′3′-c-di-GMP, revealing two oligomeric states: spiral-shaped single filaments and fiber bundles composed of straight protofibrils. In spiral filaments, the STING domain sequesters the TIR domain’s BB loop within a hydrophobic core, suppressing NADase activity. This inactive conformation is stabilized by interactions between the CBDα4 helix and the TIR domain, as well as a calcium-binding site. Conversely, fiber bundle formation—driven by inter-protofibril TIR domain interactions—disrupts these autoinhibitory contacts, liberating the BB loop to enable head-to-tail assembly of adjacent TIR domains into a composite NADase-active site. Calcium ions promote spiral filament assembly while inhibiting fiber bundles, revealing a dual regulatory role in tuning *El*STING activation. Strikingly, this mechanism diverges from single-filament systems like *Sf*STING, underscoring evolutionary diversity in STING signaling. Our findings establish distinct filament architectures as structural checkpoints governing bacterial STING activation, providing mechanistic insights into how conformational plasticity and environmental cues like calcium regulate abortive infection. These results highlight parallels between prokaryotic and eukaryotic immune strategies, emphasizing conserved principles in pathogen defense across domains of life.

**IMPORTANCE:**

Bacteria employ a sophisticated immune system, CBASS, evolutionarily related to human antiviral pathways, to defend against viral (phage) attacks. This study reveals how the bacterial protein *El*STING acts as a molecular switch, transitioning between an inactive spiral structure stabilized by calcium ions and an active fiber bundle. When calcium levels drop, *El*STING reorganizes into fiber bundles, activating its ability to degrade essential cellular molecules. This self-destructive mechanism halts phage replication by sacrificing the infected cell, protecting the bacterial population. The findings demonstrate how structural rearrangements govern life-or-death immune decisions, mirroring principles in human STING signaling. By uncovering calcium’s role in regulating this process, the work deepens our understanding of microbial immunity and highlights shared strategies across domains of life. These insights could inspire novel antimicrobial therapies or bioengineered systems to combat infections, bridging fundamental science with practical applications in health and biotechnology.

## INTRODUCTION

The cyclic GMP-AMP synthase (cGAS)-STING signaling pathway is a conserved and versatile mechanism of the innate immune system, which was originally identified in metazoans (1–4) and more recently uncovered in prokaryotes (5, 6), underscoring its evolutionary significance across domains of life (7, 8). In mammals, this pathway plays a pivotal role in fighting against cancer and viral infections (9). Similarly, in prokaryotes, it functions as an anti-phage defense mechanism in the form of the homologous cyclic oligonucleotide-based anti-phage signaling system (CBASS), a sophisticated and cell-intrinsic immune system that plays a vital role in protecting against bacteriophage infections (10, 11). Upon encountering phage infection, the core proteins of the CBASS system, cGAS/Dncv-like nucleotidyltransferases (CD-NTases), synthesize cyclic oligonucleotide signaling molecules, which are subsequently detected by CD-NTase-associated proteins (Caps), such as STING, to initiate a programmed cell suicide mechanism (12–15). This sacrificial response halts the spread of the phage by aborting the infection at the cellular level.

The cap proteins have exhibited remarkable structural and functional diversity. They are primarily characterized by two essential domains: the sensor domain that recognizes and binds the cyclic nucleotide second messengers; and the effector domain that executes the self-destructive response (16, 17) by degrading genetic material, disrupting cell membranes, or depleting essential metabolites (15, 18–22). Besides, recent studies have highlighted that ancillary Cap proteins associated with type II, III, and IV CBASS operons may also play regulatory roles, fine-tuning the anti-phage activity of CD-NTases (23–25). These accessory proteins could help modulate the sensitivity and strength of the CBASS response and potentially adjust the immune reaction to a proper degree against specific phage threats. Investigating the precise mechanisms through which these ancillary proteins interact with the CBASS system is therefore critical for the comprehensive understanding of their functionality and evolutionary progress, which may expand our knowledge of bacterial immunity and also provide insights into the evolutionary origins of innate immune pathways shared across different domains of life.

Cap12 represents a type of bacterial STING that comprises an N-terminal TIR domain and a C-terminal STING domain (10, 26–28). Prior studies have demonstrated that detection of the second messenger 3′3′-c-di-GMP triggers bacterial STING oligomerization into filaments through side-by-side packing of their STING domains (26). This interaction between neighboring STING dimers induces a rearrangement of the active sites in the TIR domain, enabling the degradation of β-nicotinamide adenine dinucleotide (NAD^+^) (26). In mammals, STING consists of an N-terminal transmembrane domain (TMD) and a C-terminal cytosolic cyclic di-nucleotide-binding domain (CBD) (29, 30). The activation of mammalian STING by the second messenger cyclic [G(2',5')pA(3',5')p] (2′3′-cGAMP) induces the formation of STING filaments on the ER membrane (30–37). The polymerized STING was then transported to ERGIC and the Golgi apparatus, and the CBD functions as a platform to recruit and activate downstream components, such as TANK-binding kinase 1 (TBK1) and the transcription factor IRF3 (34). In addition, the TMD of human STING acts as a proton channel, neutralizing post-Golgi vesicles and promoting non-canonical autophagy and cell death (30, 38, 39). In rare cases, bacterial STING may fuse with a transmembrane domain, as observed in Cap13 (10, 20, 36). However, the role of the TMD in bacterial STING during anti-phage defense remains unclear. It is uncertain whether it functions like Cap15 (21), disrupting inner membrane integrity, or serves as an ion channel similar to mammalian STING. Unlike membrane-integrated STING, bacterial STING fused with the TIR domain can form complex multi-filament architectures, as demonstrated in *Sphingobacterium faecium* TIR–STING (*Sf*STING) (26) and *Epilithonimonas lactis* TIR-STING (*El*STING) (28). However, the exact mechanisms of how these multi-filament structures regulate STING’s anti-phage activity are yet to be discovered.

In this study, cryogenic electron microscopy (cryo-EM) resolved two ligand-bound conformational states of *El*STING: an inactive spiral-shaped filament and an active fiber bundle. The spiral conformation, stabilized by calcium ions via a coordination site in the STING domain, sequesters the TIR domain’s BB loop, suppressing NADase activity. Calcium depletion disrupts these interactions, enabling a structural transition to the fiber bundle. Here, inter-protofibril TIR domain interactions mediate linear assembly, forming a composite active site through conserved head-to-tail TIR alignment, akin to other NADases such as *Sf*STING and SARM1 (26, 40). Mutagenesis and functional assays confirm the fiber bundle as the active state, linking its formation to NAD^+^ hydrolysis and anti-phage defense.

## RESULTS

### Identification of an intact CBASS system in *Epilithonimonas lactis*

**Fig 1:**
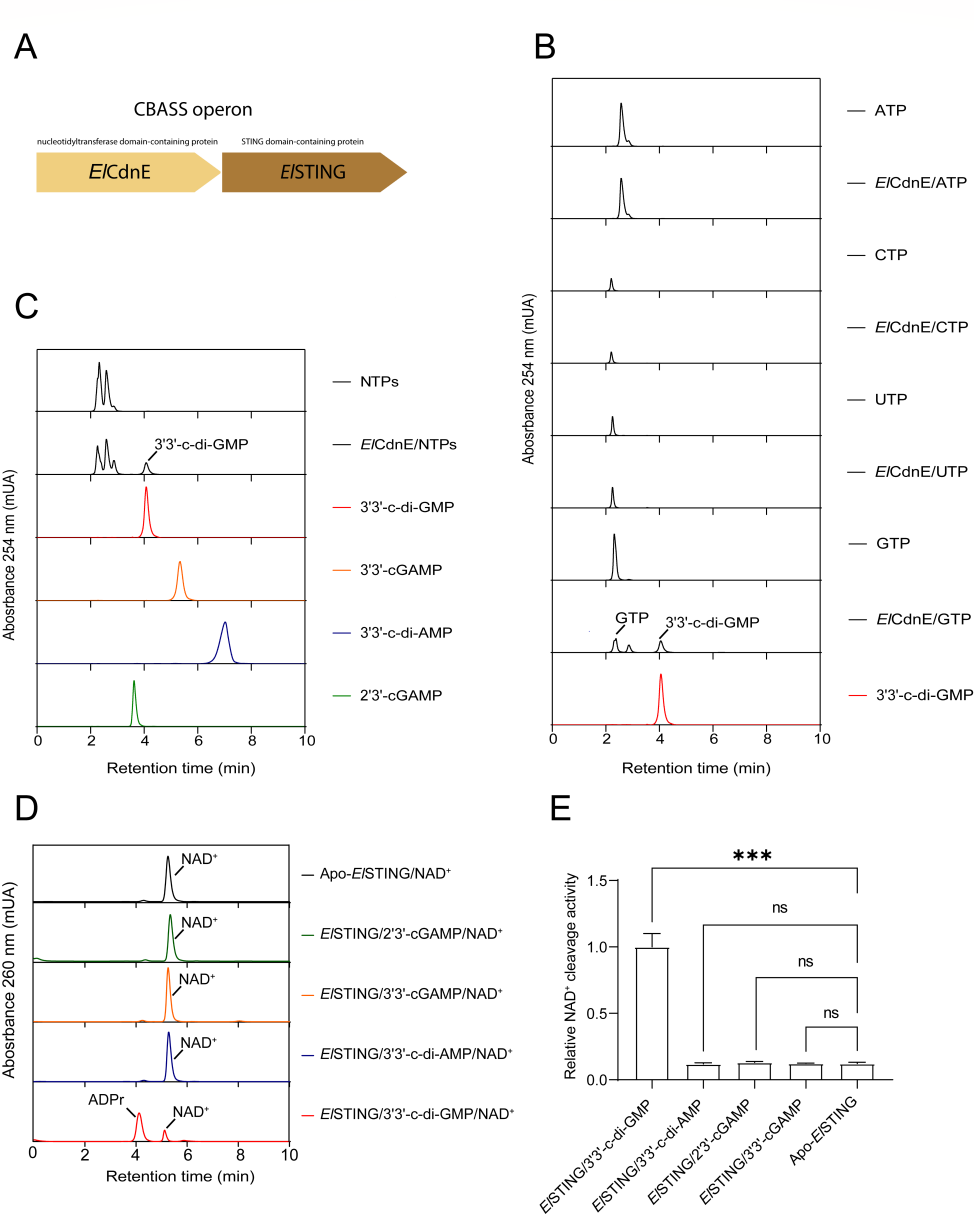
Identification of the intact cGAS-STING pathway (CBASS) in *Epilithonimonas lactis*. (**A**) Schematic representation of *E. lactis El*CdnE (nucleotidyltransferase domain-containing protein) and *El*STING (STING domain-containing protein). Domain architectures of *El*CdnE and *El*STING are shown. (B–C) HPLC analysis of *El*CdnE substrate preference. Substrate preference of *El*CdnE analyzed by HPLC. ATP, CTP, UTP, or GTP was incubated with or without *El*CdnE. Absorbance traces at 254 nm (mAU) demonstrate *El*CdnE-dependent synthesis of 3′3′-c-di-GMP from GTP (**B**). Specificity of *El*CdnE for cyclic dinucleotide production. *El*CdnE was incubated with NTPs (ATP, GTP, CTP, or UTP), and reaction products were resolved by HPLC. 3′3′-c-di-GMP, 3′3′-c-di-AMP, 3′3′-cGAMP, and 2′3′-cGAMP standards are included for comparison (**C**). (D–E) Ligand specificity of *El*STING assessed by NAD^+^ cleavage activity. Ligand-dependent *El*STING NADase activity assessed by HPLC. Apo-*El*STING or *El*STING pre-incubated with 3′3′-c-di-AMP, 3′3′-cGAMP, 3′3′-c-di-GMP, or 2′3′-cGAMP was reacted with NAD^+^. Absorbance traces show NAD^+^ consumption and product formation (**D**). Quantification of relative NAD^+^ cleavage activity (mean ± SD). Statistical significance was calculated using a two-tailed Student’s *t*-test with ***P < 0.001 (**E**).

Although previous studies identified 3′3′-c-di-GMP as the preferred ligand of *El*STING (WP_034976296.1), the presence of a CD-NTase capable of synthesizing this cyclic dinucleotide within the same operon remained unconfirmed. A detailed analysis of the genomic context surrounding *El*STING revealed an upstream gene (WP_034976293.1) encoding a nucleotidyltransferase domain-containing protein (CD-NTase) (Fig. 1A). Based on its homology to CdnE enzymes within CdnE-TIR-STING CBASS operons (14), we designated this protein *El*CdnE and hypothesized that it synthesizes the 3′3′-c-di-GMP ligand required for *El*STING activation.

To determine the catalytic specificity of *El*CdnE, we performed *in vitro* enzymatic assays using ATP, GTP, UTP, CTP, or mixed NTPs as substrates, followed by high-performance liquid chromatography (HPLC) to characterize the reaction products. *El*CdnE exhibited exclusive specificity for GTP, catalyzing the synthesis of 3′3′-c-di-GMP, with no detectable production of other cyclic dinucleotides (e.g., 3′3′-c-di-AMP or 3′3′-cGAMP) (Fig. 1B and C). These results confirm the presence of a functional CBASS operon in *Epilithonimonas lactis*, wherein *El*CdnE produces 3′3′-c-di-GMP to activate the downstream effector *El*STING.

Intriguingly, size exclusion chromatography (SEC) revealed that *El*STING undergoes ligand-induced oligomerization in the presence of 3′3′-c-di-GMP, 3′3′-c-di-AMP, or 3′3′-cGAMP, but not 2′3′-cGAMP (Fig. S1A). Consistent with this, negative-stain microscopy demonstrated that 3′3′-cyclic di-nucleotides (CDNs)—though not 2′3′-cGAMP—promote the assembly of *El*STING into long spiral-shaped filaments (Fig. S1B). Strikingly, 3′3′-c-di-GMP induced an additional structural phenotype, driving the formation of fiber bundles alongside spiral filaments as observed in a previous study (28). Functional assays confirmed that 3′3′-c-di-GMP is the sole CDN capable of activating *El*STING-mediated NAD^+^ degradation (Fig. 1D and E), cleaving NAD^+^ into ADP-ribose (ADPr) and nicotinamide (NAM) (Fig. S1C) as observed in *Sf*STING homologs (20). This ligand specificity underscores 3′3′-c-di-GMP as the physiological activator of *El*STING. The observed structural heterogeneity (spiral filaments vs. fiber bundles) suggests distinct oligomeric states may regulate *El*STING’s signaling output, potentially linking filament morphology to enzymatic activation.

### Structure of *El*STING/3′3′-c-di-GMP single filament

To investigate the functional relevance of the two *El*STING filament morphologies, we employed cryo-electron microscopy (cryo-EM) to resolve their structures at near-atomic resolution. The purified recombinant *El*STING was incubated with 3′3′-c-di-GMP overnight at 4°C. The sample was then applied to freshly glow-discharged cryo-EM grids, blotted, and rapidly vitrified in liquid ethane to preserve native filament architectures. Cryo-EM analysis revealed that 3′3′-c-di-GMP induces *El*STING to assemble into two distinct oligomeric states: spiral-shaped filaments and tightly packed fiber bundles (Fig. S2 and S3), as observed by negative-stain microscopy (Fig. S1B). Both architectures were consistently observed across multiple cryo-EM data sets, confirming that ligand binding drives conformational plasticity in *El*STING. The spiral filaments exhibit a helical arrangement with periodic subunit interfaces, while fiber bundles adopt a parallel, higher-order packing mode.

**Fig 2:**
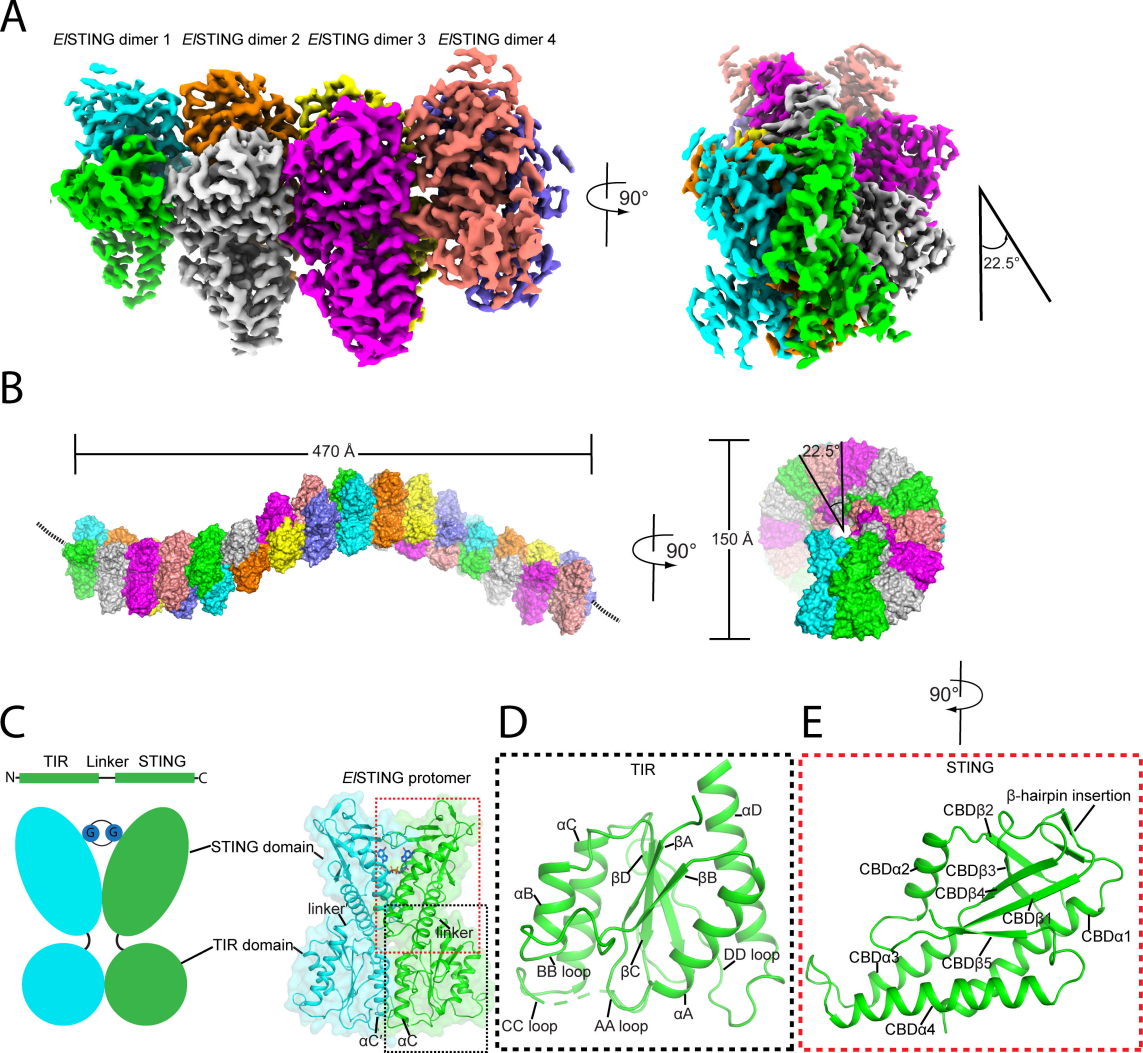
Overall structure of the spiral-shaped *El*STING/3′3′-c-di-GMP filament. (**A**) Cryo-EM map of the spiral-shaped *El*STING octamer, showing the arrangement of *El*STING dimers (Dimer 1–Dimer 4) along the polymer axis with a rotational alignment of 22.5°. Side and top-down views illustrate the helical architecture. (**B**) The spiral-shaped architecture of the *El*STING/c-di-GMP filament forms a solenoid-like structure. The left panel shows the elongated side view, with overall dimensions labeled (470 Å length, 150 Å width). The right panel highlights the helical symmetry with a rotation of 22.5° between adjacent subunits. (**C**) Schematic representation of *El*STING’s domain organization, including the TIR and STING domains connected by a linker. Right panel: Surface representation of the *El*STING protomer with key regions labeled. (**D and E**) Cartoon representations of the TIR domain (D) and STING domain (E), with secondary structural elements labeled (e.g., loops and helices) as referenced in the text.

The structure of the *El*STING/3′3′-c-di-GMP spiral-shaped filament was determined at 2.88 Å resolution (Fig. S2 and S4A). Unlike the straight configuration observed in the *Sf*STING/3′3′-c-di-GMP filament (26), the *El*STING single filament adopts a spiral configuration, with adjacent STING dimers rotating approximately 22.5° and forming 16 dimers per turn (Fig. 2A and B). From a top-down perspective, the *El*STING filament appears as a solenoid with a diameter of 150 nm, while the side view reveals a turn length of 470 Å (Fig. 2B). The overall structure of the *El*STING dimer closely resembles that of the *Sf*STING dimer, comprising an N-terminal TIR domain and a C-terminal STING domain, connected by a linker (Fig. 2C). The TIR domain adopts an α/β fold with four central parallel β-strands (βA, βB, βC, and βD), which is surrounded by four helices (αA, αB, αC, and αD), and features four loops (AA loop, BB loop, CC loop, and DD loop) (Fig. 2D). The STING domain, also referring to the cyclic di-nucleotide binding domain (CBD), adopts the classical STING fold characteristic of mammalian STING with an additional small extra β-hairpin insertion between CBDα1 and CBDβ1 and the lack of the C-terminal helix in mammalian counterparts (Fig. 2E). The two TIR domains are arranged in a back-to-back configuration, with their αC helices packing against each other (Fig. 2C). The two STING domains form a cyclic di-nucleotide binding pocket that engages 3′3′-c-di-GMP in a symmetric manner (Fig. 2C). The linker regions in the *El*STING dimer bind 3′3′-c-di-GMP in a parallel orientation (Fig. 2C), similar to what is observed in *Sf*STING (26).

**Fig 3:**
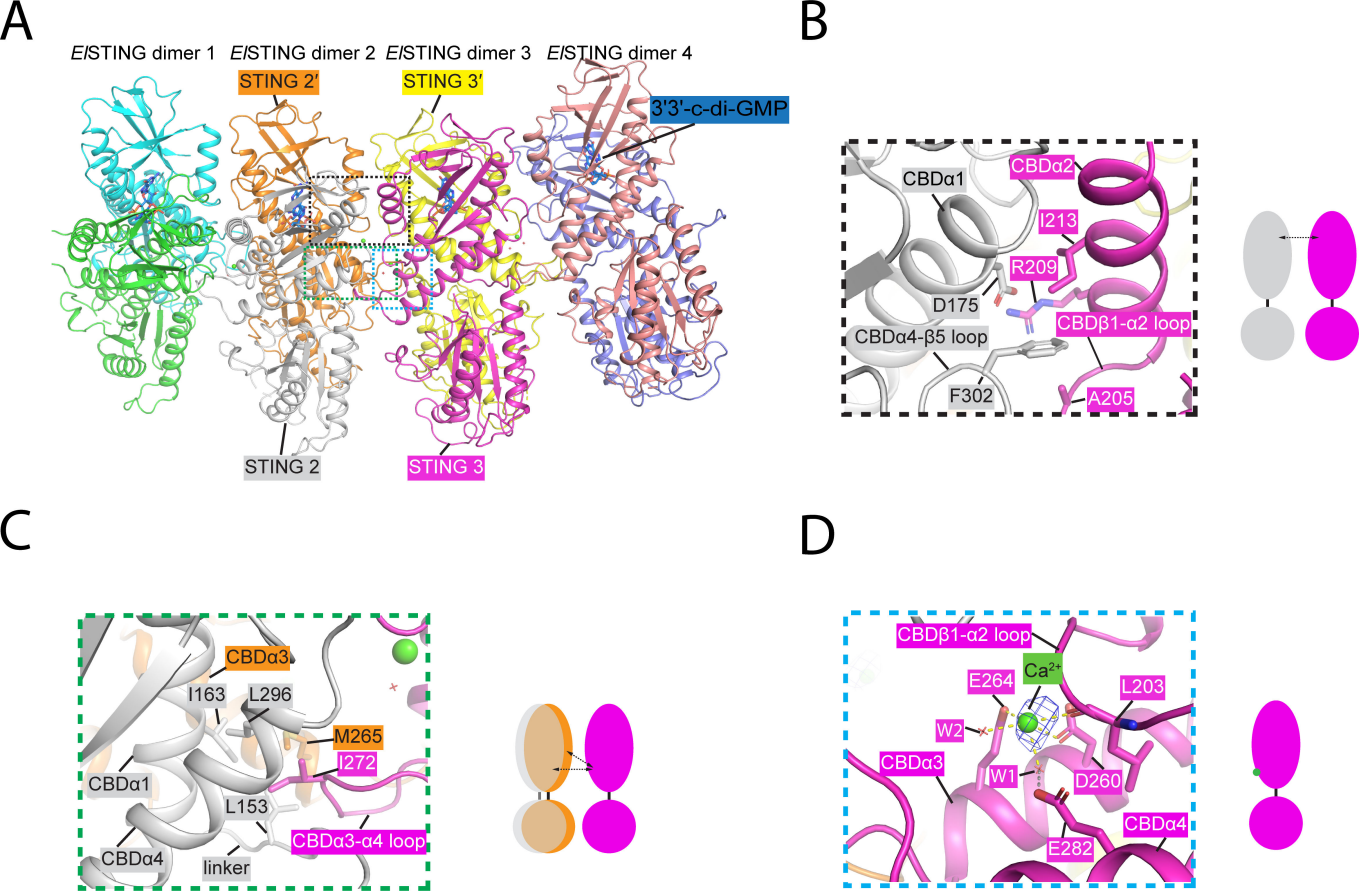
Detailed structure analysis of the spiral-shaped *El*STING/3′3′-c-di-GMP filament. (**A**) Cartoon depiction of the *El*STING octamer in a spiral-shaped assembly. Individual dimers are displayed in different colors to highlight their relative positions. (**B**) Detailed view of the upper interaction site between STING domains, with key residues involved in the interaction labeled. (**C**) Enlarged view of the lower interaction site, showing residues mediating inter-dimer interactions. (**D**) Close-up view of the calcium-binding site. The calcium ion is shown as a green sphere, with coordination bonds represented by yellow dashed lines and hydrogen bonds by gray dashed lines. The contour level = 6σ.

A notable feature of the *El*STING spiral-shaped filament is that its formation is exclusively mediated by interactions between adjacent STING domains, and the N-terminal TIR domain does not participate in oligomerization (Fig. 2A and 3A). The STING-STING interactions in the *El*STING oligomer involve two critical regions. The upper interaction site is stabilized by hydrophobic interactions between residue F302 in CBDα4-β5 loop of STING1 and residue A205 in CBDβ1-α2 loop and residue I213 in CBDα2 of STING2, along with a salt bridge formed between residue D175 in CBDα1 of STING1 and residue R209 in CBDα2 of STING2 (Fig. 3B). The lower interaction site features a loop (CBDα3-α4 loop) that inserts into a hydrophobic pocket formed by the neighboring STING dimer and the linker connecting the STING and TIR domains. Specifically, residue I272 in the CBDα3-α4 loop of STING3 forms hydrophobic interactions with residues I163 and L296 in CBDα1 and CBDα4, respectively, of STING2, as well as with residue M265 in CBDα3 of STING2′ and residue L153 in the linker region of STING2 (Fig. 3C).

Interestingly, the cryo-EM density map revealed a prominent ion-coordination site near the lower interaction interface of the *El*STING spiral. This site is defined by a coordination sphere comprising the side chains of two acidic residues (D260 and E264) in the CBDα3 helix, the backbone carbonyl group of L203 within the CBDβ1-α2 loop, and two water molecules (Fig. 3D). One of these water molecules is further hydrogen-bonded to E282 in the CBDα4 helix, completing a coordination geometry of ion binding. Based on the observed coordination chemistry and the physiological prevalence of calcium as a cofactor, we tentatively assigned this density to a calcium ion (Ca²^+^). The Ca²^+^ likely co-purifies with *El*STING under native conditions, suggesting it binds endogenously and may play a structural or regulatory role in oligomer stabilization. This finding aligns with observations in the crystal structure of human STING, where Ca²^+^ was identified (41, 42). The resemblance between the Ca²^+^-binding sites in *El*STING and human STING structures highlights the evolutionary conservation of the STING molecule, supporting the hypothesis of a bacterial origin for mammalian STING (5).

A previous structural study has revealed that the STING domain of *El*STING adopts a spiral-shaped assembly when bound to 3′3′-c-di-AMP, propagating through the crystal lattice in a manner analogous to the architecture observed in the spiral-shaped *El*STING/3′3′-c-di-GMP complex (28) (Fig. 3A; Fig. S5A). Notably, the crystal structure of the *El*STING/3′3′-c-di-AMP complex showed no evidence of bound calcium ions, suggesting that calcium may not be required for 3′3′-c-di-AMP-induced oligomerization (28). This conclusion is further supported by negative-stain electron microscopy (EM) studies demonstrating that EDTA, a calcium-chelating agent, fails to disassemble the spiral-shaped filaments or induce a transition to the fiber bundle architecture in both the *El*STING/3′3′-c-di-AMP and *El*STING/3′3′-cGAMP complexes (Fig. S1B). Nevertheless, the mechanism underlying the inability of 3′3′-c-di-AMP and 3′3′-cGAMP to promote *El*STING fiber bundle formation remains unresolved and warrants further investigation.

### Structure of *El*STING/3′3′-c-di-GMP fiber bundle

**Fig 4:**
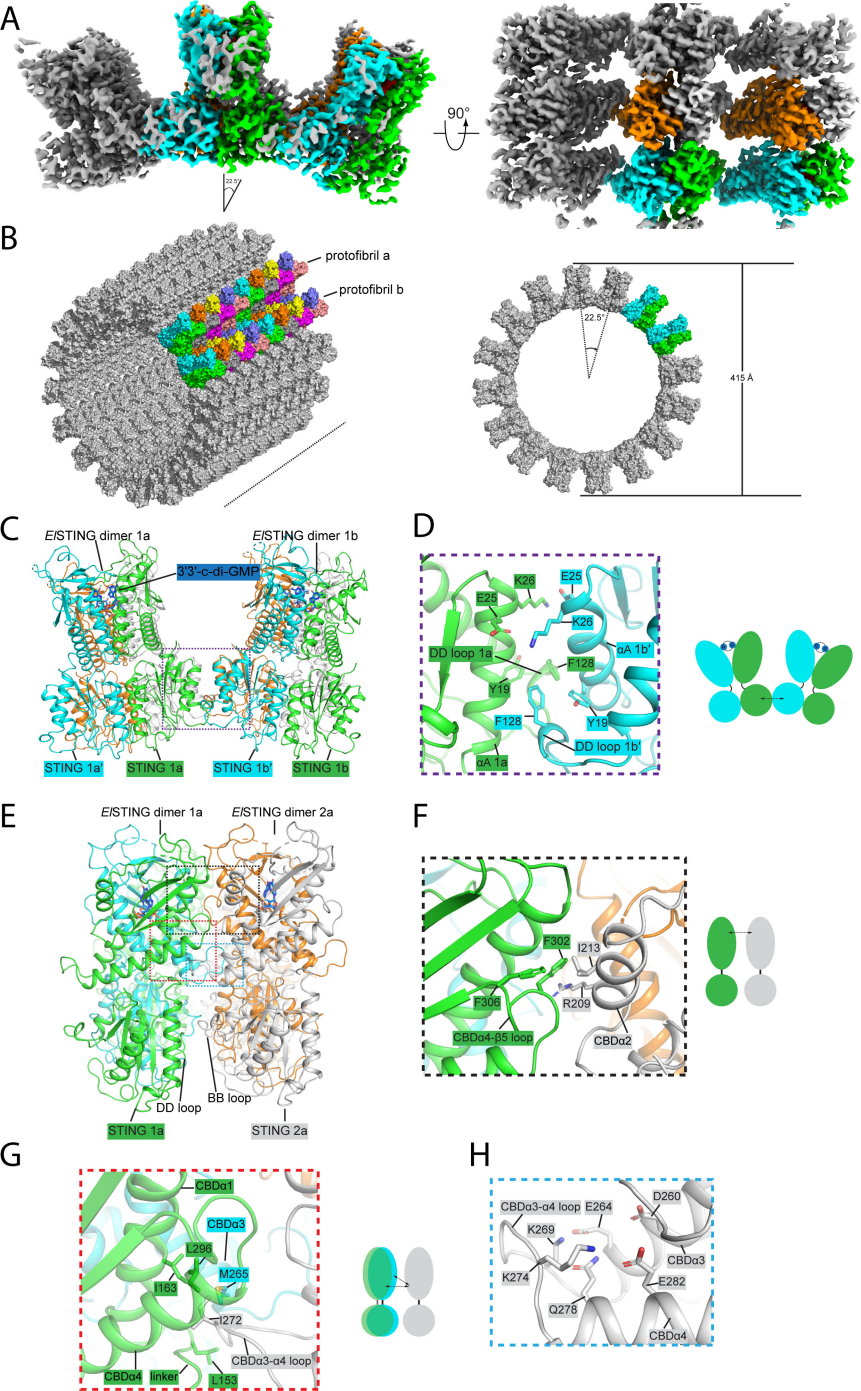
Structure of the *El*STING/3′3′-c-di-GMP fiber bundle. (**A**) Cryo-EM map of the *El*STING/3′3′-c-di-GMP fiber bundle, showing the arrangement of protofibrils. The rotational alignment of one protofibril relative to the adjacent protofibril is indicated. Left panel: side view of the fiber bundle. Right panel: top-down view highlighting the helical symmetry of the assembly. (**B**) The fiber bundle architecture of the *El*STING/3′3′-c-di-GMP complex forms a hollow columnar structure. The left panel displays the oblique view of the fiber bundle, and the right panel shows the top-down view, emphasizing the circular arrangement with a 22.5° rotation between subunits and an outer diameter of 415 Å. (**C**) Cartoon representation of adjacent *El*STING protofibrils, illustrating their structural arrangement. (**D**) Left panel: enlarged view showing the interactions between TIR domains of adjacent *El*STING protomers. Key residues (e.g., Y19, E25, K26, F128) involved in these interactions are labeled. Right panel: schematic representation of interactions between adjacent protofibrils. (**E**) Cartoon depiction of two *El*STING dimers within the fiber bundle. (**F**) Enlarged view of the upper interaction site, showing residues (e.g., I213, F306, R209) contributing to the CBDα4-β5 loop interactions. (**G**) Enlarged view of the lower interaction site, highlighting residues (e.g., L153, I163, M265) involved in interactions between the linker region, CBDα3-α4 loop, and adjacent domains. Schematic representations of the interactions are provided below each panel for clarity. (**H**) View of the disrupted calcium-binding site, revealing the rearrangement of residues near the previously identified calcium-binding region, reflecting structural adaptation.

3′3′-c-di-GMP induces *El*STING to form a higher-order oligomerization in the form of fiber bundles, in addition to the previously described single filaments. The structure of the fiber bundle was resolved at 3.7 Å resolution (Fig. S3 and S4B). The packing of the *El*STING fiber bundle is mediated by the TIR domain, with each protofibril rotating approximately 22.5° to its neighboring protofibril (Fig. 4A and B). The lateral extension of these protofibrils would result in a closed circle, forming a hollow *El*STING column with a cylindrical structure approximately 415 Å in diameter (Fig. 4B). In contrast to the spiral architecture of *El*STING single filaments, protofibrils within the *El*STING fiber bundle adopt a straight configuration (Fig. 4), mirroring the linear assembly observed in *Sf*STING filaments (Fig. S5A). Notably, the interactions between the TIR domains of protofibrils contribute to their assembly (Fig. 4C). Specifically, F128 within the DD loop of one TIR domain packs against residues Y19 and F128 of a neighboring domain. This interaction is further stabilized by electrostatic forces between E25 and K26 in the αA helices of adjacent TIR domains (Fig. 4C and D). A similar mechanism governs inter-filament interactions in the *Sf*STING/3′3′-c-di-GMP double fiber, where the DD loop of the TIR domain facilitates fiber formation (26), suggesting a conserved assembly mechanism across these systems. However, a notable divergence arises in *Sf*STING/3′3′-c-di-GMP double fiber: the interaction partner is the STING domain of an adjacent *Sf*STING filament, rather than another TIR domain. This distinction highlights a key variation in the structural organization of these assemblies.

**Fig 5:**
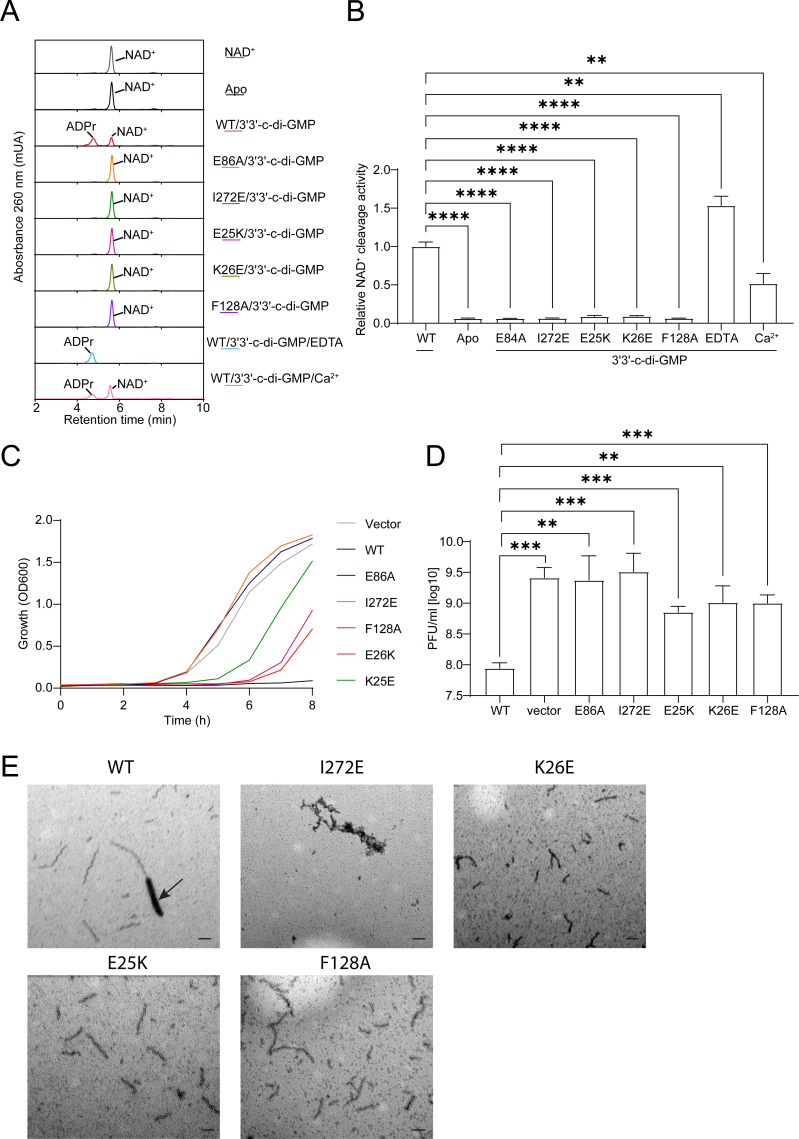
Enzymatic activity of *El*STING and its variants. (**A**) Representative HPLC chromatograms illustrating the enzymatic products of *El*STING. (**B**) Relative NAD^+^ cleavage activity of *El*STING mutants and under different conditions (EDTA and Ca²^+^), compared to the wild-type (WT). Statistical significance was calculated using a two-tailed Student’s t-test, with **P < 0.01 and ****P < 0.0001. Data are presented as mean ± S.D. from three independent biological replicates (*n* = 3). (**C**) Cell toxicity assessment of WT *El*STING and its variants, evaluated through bacterial growth assays. The initial OD_600_ of the bacterial culture is provided as a reference. (**D**) Phage replication assay in *E. coli* expressing WT *El*STING and its variants, quantified by plaque-forming units (PFU). Statistical significance was calculated using a two-tailed Student’s *t*-test, with **P < 0.01 and ***P < 0.001. Data are presented as mean ± S.D. from three independent biological replicates (*n* = 3). (**E**) Negative-stain EM micrographs for wt and mutants in the presence of 3′3′-c-di-GMP. The arrow indicates the fiber bundle of *El*STING induced by 3′3′-c-di-GMP. Scale bar = 100 nm. Images representative of *n* ≥ 3 experiments.

To investigate the physiological significance of the *El*STING fiber bundle, mutagenesis studies were performed. Mutants E25K, K26E, and F128A, designed to disrupt TIR interactions, were analyzed, as well as the I272E mutant to disrupt STING-STING interactions. The catalytic residue E86, which is essential for enzymatic activity, was mutated to alanine (E86A) to generate a catalytically dead control. The mutants were purified, with their enzymatic activity assessed via HPLC. In the presence of 3′3′-c-di-GMP, wild-type *El*STING degraded NAD^+^, generating the product (ADPr) with a short retention time on the HPLC column (Fig. 5A and B). By contrast, the E86A and I272E mutants seemed to lose their ability to degrade NAD^+^, as no product has been detected (Fig. 5A and B). Nonetheless, the E25K, K26E, and F128A mutants exhibited extremely low enzymatic activity, producing markedly less NAD degradation product compared to wild-type *El*STING (Fig. 5A and B). Next, cell toxicity assays were performed to examine whether the mutants would attenuate cytotoxic effects. Compared to wild-type *El*STING, the E25K, K26E, and F128A mutants displayed reduced cytotoxicity toward *E. coli*, while the E86A and I272E mutants have completely lost their cytotoxicity (Fig. 5C), consistent with the HPLC results.

Finally, the importance of the *El*STING fiber bundle in defending against bacteriophage infection was assessed. The BL21(DE3) pLysS strains expressing wild-type or mutant *El*STING were inoculated with T2 phage, and the phage culture titers were measured after 8 h. The results revealed that *E. coli* expressing wild-type *El*STING could effectively inhibit phage proliferation, whereas the *E. coli* expressing TIR-disrupting mutants exhibited reduced inhibition, in particular, with the strains expressing the I272E or E86A mutant showing up to no effect on phage proliferation (Fig. 5D). Consistent with these findings, negative-stain TEM analysis revealed that mutants disrupting TIR domain interactions (E25K, K26E, F128A) failed to assemble into fiber bundles under 3′3′-c-di-GMP stimulation, instead forming exclusively spiral-shaped filaments (Fig. 5E). By contrast, the I272E mutant failed to oligomerize, likely adopting a dimeric state. Together, these results confirm that the *El*STING fiber bundle is indispensable for its NADase activity, host cytotoxicity, and antiphage infection.

### Structural comparison between the spiral-shaped *El*STING filament and the *El*STING fiber bundle

The transition of the *El*STING fibril from spiral to straight configuration represents the most significant structural rearrangement of the *El*STING/3′3′-c-di-GMP complex. During fiber bundle formation, STING-STING interactions induce a rotational shift in *El*STING (Fig. 2B and 4B), triggering subsequent rearrangements of interfacial residues in both the upper and lower domains. Specifically, residue F302 in the CBDα4-β5 loop of STING1 would move inward and pack against residue R209 in the CBDα2 of STING2 (Fig. 4E and F). Meanwhile, residue I213 in CBDα2 of STING2 would insert into a hydrophobic pocket formed by residues F302 and F306 in CBDα4-β5 loop of STING1 (Fig. 4F). Although the hydrophobic interaction in the lower region remains largely unchanged, the CBDα3-α4 loop shifts upward compared to its position in the spiral-shaped structure (Fig. 4G).

**Fig 6:**
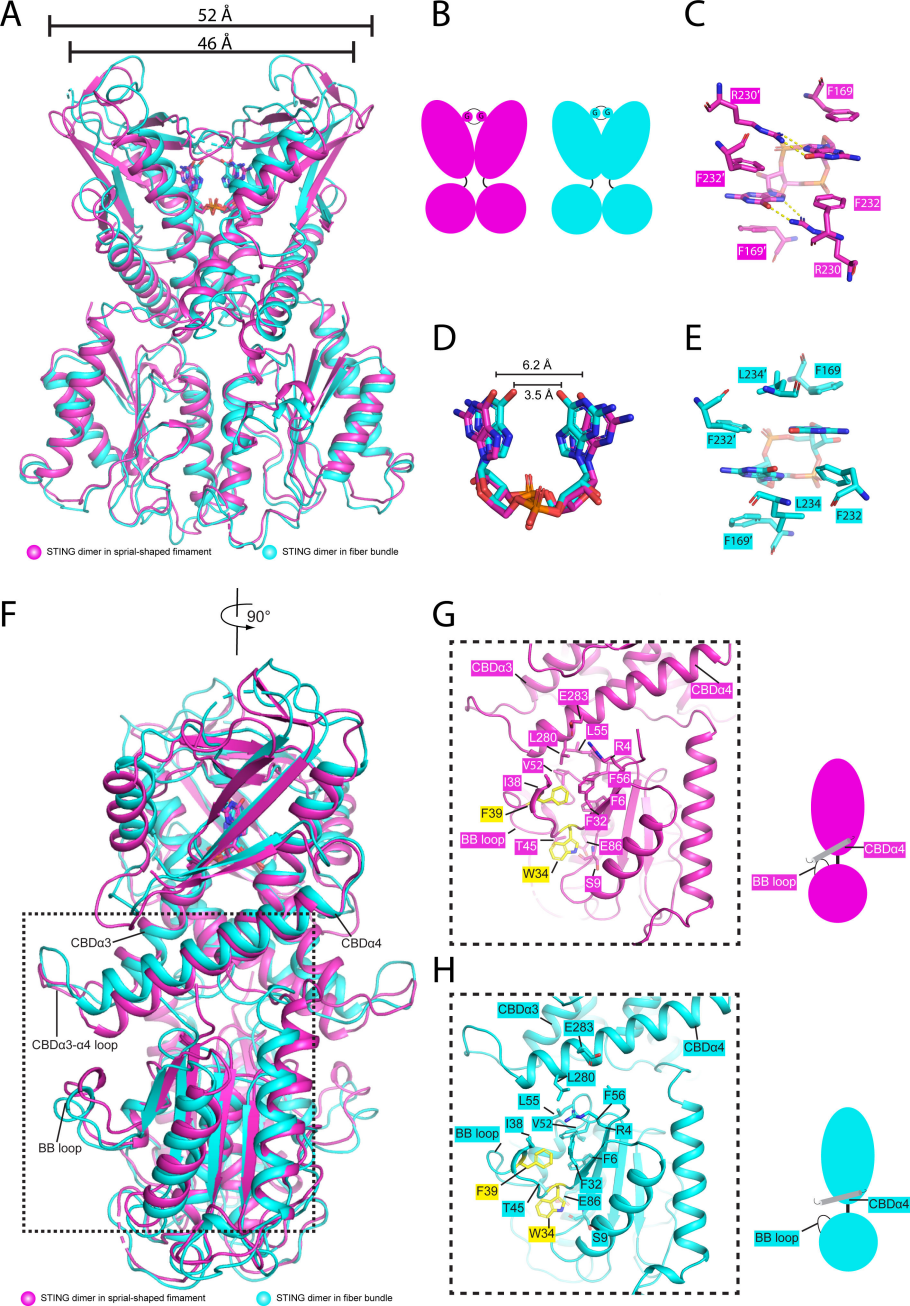
Structural comparison of *El*STING dimers from the spiral-shaped filament and fiber bundle. (**A, B, and F**) Superimposition of the *El*STING dimer from the spiral-shaped filament (magenta) and fiber bundle (cyan), shown as cartoons. The dimensions (52 Å and 46 Å) between the tips of the protomers are labeled. Panel B provides a schematic representation of the superimposed structures, emphasizing structural similarities and differences. (**C, E**) Interaction networks between 3′3′-c-di-GMP and *El*STING. Residues involved in 3′3′-c-di-GMP binding are displayed in stick representations. Hydrogen bonds are represented by yellow dashed lines. Panel C highlights key binding residues in the spiral-shaped filament (magenta), while panel E shows equivalent interactions in the fiber bundle (cyan). (**D**) Superimposition of 3′3′-c-di-GMP molecules from the spiral-shaped filament and fiber bundle structures. The distances between the carbonyl moieties (6.2 Å and 3.5 Å) are indicated. (**G and H**) Detailed views of the interactions between the BB loop and CBDα4 in the spiral-shaped *El*STING (magenta, panel G) and fiber bundle *El*STING (cyan, panel H). Key residues involved in these interactions (e.g., W34, F39, T45, L280, E86) are labeled. Schematic representations on the right illustrate the spatial relationship between the BB loop and CBDα4 in each structure.

Superimposition of *El*STING dimers from the spiral-shaped filament and fiber bundle reveals striking structural divergences. First, the distance between the two STING tips decreases, measuring 52 Å in the spiral-shaped STING and 46 Å in the STING fiber bundle (Fig. 6A), and the four β-strands forming the lid region move closer together, resulting in a smaller 3′3′-c-di-GMP binding pocket in the fiber bundle structure (Fig. 6B). Second, the 3′3′-c-di-GMP binding mode differs significantly between these two structures. In the spiral-shaped structure, residue R230 recognizes the Hoogsteen edge of the guanine ring of 3′3′-c-di-GMP, packing against residue F232; simultaneously, residues F232 and F169 form a four-layer binding mode, which is commonly observed in other bacterial STING/3′3′-c-di-GMP complexes (26–28) (Fig. 6C). By contrast, in the protofibril structure, the two guanine rings move closer, transforming the U-shaped 3′3′-c-di-GMP into a Δ-shaped conformation (Fig. 6D). Here, the distance between the carbonyl moieties of the purine rings is reduced to approximately 3.5 Å, compared to about 6.2 Å in the spiral-shaped structure (Fig. 6D). Consequently, the interaction between the guanine ring and residue R230 is lost, with residue F232 occupying its position and aligning with the Hoogsteen edge of the guanine ring. In addition, residue L234 is observed packing against the guanine ring, resulting in a Leu/Phe/Guanine/Phe four-layer binding mode which replaces the Arg/Phe/Guanine/Phe mode, seen in the spiral-shaped structure (Fig. 6E). Lastly, due to inter-protofibril interactions, the TIR domain undergoes a notable rotation along the dimer axis compared to its spiral-shaped conformation (Fig. 2B and 4B), which relocates the N-terminus of CBDα4 and elevates the CBDα3-α4 loop, contributing to the structural rearrangements (Fig. 6F).

Structural alignment of the *Sf*STING dimer with *El*STING dimers from spiral-shaped filaments and fiber bundles revealed distinct conformational differences (Fig. S5B). The *El*STING fiber bundle dimer aligns more closely with *Sf*STING, exhibiting a lower root mean square deviation (RMSD) of 2.1 Å (vs. 3.4 Å for the spiral-shaped dimer). This similarity is particularly evident in the STING domain, where the inter-tip distances (47 Å for *Sf*STING, 46 Å for *El*STING fiber bundle, and 52 Å for spiral-shaped *El*STING) and c-di-GMP binding conformations nearly overlap (Fig. S5B). These findings suggest that bacterial STING activation involves a contraction of the STING domain—a mechanism previously observed in human STING activation—implying evolutionary conservation of this structural rearrangement from bacteria to mammals (35).

### Structural insights into catalytic site remodeling

To investigate how these structural changes drive *El*STING activation, catalytic site differences were analyzed in detail. The conserved residues W34 and F39 in the BB loop are believed to bind the ribose and nicotinamide moieties, similar to their role in other TIR NADases (40, 43–45). In the spiral-shaped structure, the side chain of residue W34 is shielded by the carbonyl group of residue S9 and the side chain of residue T45 (Fig. 6G). Most importantly, residue F39 is buried in a hydrophobic core formed by residues F6, F32, I38, F56, V52, and L55 from the TIR domain, as well as residue L280 from the CBDα4 helix of the STING domain; a salt bridge exists between residue R4 from the TIR domain and residue E283 from the CBDα4 helix (Fig. 6G). These interactions mutually stabilize and fix the residue F39 interaction network, effectively burying the substrate-binding residues and hence preventing NAD^+^ association. Importantly, NADase activation requires head-to-tail assembly of adjacent TIR domains via the BB loop in BE (termed BD interface in TIR-STING) interface (44, 45). However, in the spiral conformation, rotation of the TIR domain along the dimer axis prevents the BB loop from engaging the DD loop of a neighboring TIR domain (Fig. S5C), thereby blocking formation of a composite active site. These features strongly support the conclusion that the spiral-shaped conformation represents *El*STING’s inactive state (Fig. S5C). By contrast, the fiber bundle conformation induces marked catalytic site reorganization. Here, the carbonyl group of S9 and the side chain of T45 no longer occlude W34 (Fig. 6H). Although F39 remains buried, upward displacement of the CBDα4 helix disrupts its interactions with the hydrophobic core (Fig. 6H), likely permitting NAD^+^ binding to trigger F39 flipping and active site assembly. Notably, the structural architecture of *El*STING protofibrils closely resembles that of active *Sf*STING filaments, with adjacent TIR domains adopting the conserved head-to-tail assembly characteristic of the activated state (Fig. S5C). A critical distinction, however, lies in the BB loop conformation: in *El*STING protofibrils, the BB loop (“head”) remains disengaged from the DD loop (“tail”) of adjacent TIR in the BD interface (Fig. 4E; Fig. S5C), requiring substrate-induced rearrangement to establish functional interactions. By contrast, in *Sf*STING, the BB loop is pre-organized into an active configuration through direct contact with the DD loop of the adjacent TIR in the BD interface (Fig. S5C). Collectively, these structural and mechanistic parallels confirm that the *El*STING fiber bundle represents its active form, underscoring the role of conformational dynamics in STING-mediated activation.

### The inhibition of calcium ions on the fiber bundle formation

This upward shift of the CBDα4 region within the fiber bundle disrupts the calcium-binding site observed in the spiral-shaped structure (Fig. 3D and 4H). Notably, residue Q278 occupies the site which was previously held by the calcium ion, while residues K269 and K274 establish charge-charge interactions with E264 and E282, respectively. These findings suggest that calcium ions play an inhibitory role in fiber bundle formation, thereby restraining the *El*STING’s NADase activity. To test this hypothesis, we examined the NADase activity of *El*STING in the presence of calcium or chelating reagent ethylenediaminetetraacetic acid (EDTA). The inclusion of EDTA in the reaction buffer allowed *El*STING to completely degrade NAD^+^ (Fig. 5A and B), whereas the presence of calcium ions reduced its activity by half compared to that activated by 3′3′-c-di-GMP alone (Fig. 5A and B). While size-exclusion chromatography (SEC) showed that EDTA or calcium did not alter the oligomeric state of *El*STING upon 3′3′-c-di-GMP stimulation (Fig. S1A), negative-stain EM revealed striking structural differences: EDTA dramatically enhanced 3′3′-c-di-GMP-induced fiber bundle assembly, whereas calcium promoted the formation of elongated spiral-shaped filaments (Fig. S1B). Notably, EDTA failed to induce fiber bundle formation when *El*STING was stimulated by 3′3′-c-di-AMP, 3′3′-cGAMP, or 2′3′-cGAMP (Fig. S1B). These findings reinforce the hypothesis that the *El*STING fiber bundle conformation represents its active state, directly linked to its functional activation.

### Evolutionary conservation of calcium-mediated inhibition and divergent activation mechanisms in bacterial TIR-STING homologs

As demonstrated above, *El*STING inhibition is mediated by calcium binding and the interaction between its CBD α4 helix (especially, residue L280) and the TIR domain. To determine whether these inhibitory mechanisms are evolutionarily conserved across bacterial TIR-STING homologs, we performed an NCBI database search to identify functionally related proteins. Phylogenetic analysis placed *El*STING within a clade comprising 14 homologous sequences (Fig. S6). Sequence alignment demonstrated strong conservation of key residues—D260, E264, L203, and L280—indicating that these TIR-STINGs likely utilize analogous structural mechanisms: spiral-shaped inhibitory filaments and activated fiber-bundle conformations. Notably, these residues are not conserved in *Sf*STING, which adopts a single filament as its active form and occupies a distinct phylogenetic clade. This divergence underscores mechanistic differences between TIR-STING systems that employ fiber bundle architectures and those like *Sf*STING, which rely on a single filament for activation.

## DISCUSSION

The Cap proteins in the CBASS of prokaryotes play a pivotal role in inducing programmed cell death to prevent phage replication through abortive infection (11). This self-destructive mechanism ensures the survival of the bacterial population. Understanding the regulation of these Cap proteins is one of the key pieces for the complete puzzle of CBASS. Previous studies have demonstrated that TIR-STING, a critical type of Cap protein, requires 3′3′-c-di-GMP-induced filament formation to activate the NADase function of its TIR domain (20). While previous studies have identified double filaments in *Sf*STING and fiber bundles in *El*STING (26–28), the generality of the regulatory mechanism unveiled by the *Sf*STING filament structure requires further investigation.

**Fig 7:**
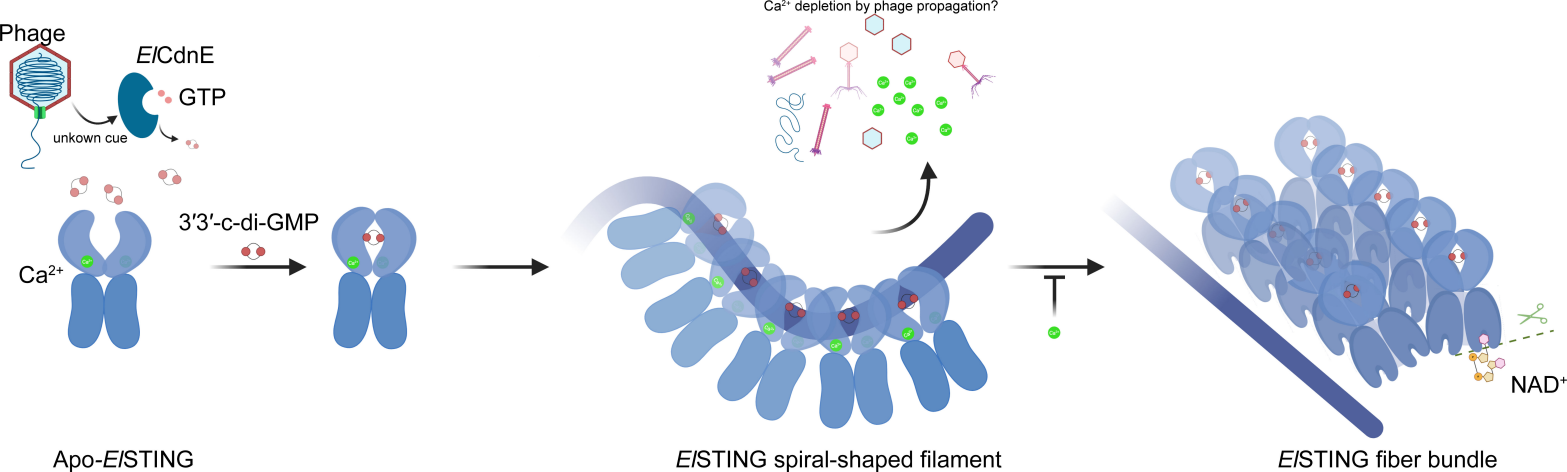
Proposed activation model for *El*STING. The model illustrates the transition from the apo state to the active polymeric state upon binding 3′3′-c-di-GMP, triggering conformational changes and subsequent activation of NADase activity. The inhibitory role of calcium ion is also shown.

In this study, cryo-EM was primarily utilized to investigate the assembly mechanism of the *El*STING oligomer. Surprisingly, the results have shown that the spiral-configured *El*STING single filament is inactive, whereas fiber bundle formation is essential for the activation of *El*STING (Fig. 7), which contrasts with the single-filament activation mechanism observed in *Sf*STING reported in a previous study (26). Through a series of structural analyses, the spiral-shaped structure has been shown to exhibit strong interactions between the N-terminus of helix CBDα4 in the STING domain and the BB loop in the TIR domain. These interactions effectively sequester key NAD^+^ recognition residues, such as F39, thereby preventing the formation of the active site. In the protofibril structure, however, such interactions are disrupted, which allows the BB loop to undergo substrate-induced rearrangements, enabling NAD^+^ binding and thus the formation of the active site. Furthermore, in the spiral-shaped *El*STING conformation, the misalignment of the TIR domain along the dimer axis sterically hinders the BB loop from engaging the DD loop of a neighboring TIR domain in the BD interface, thereby blocking formation of the composite active site (Fig. S5C). By contrast, the STING straight filament aligns adjacent TIR domains in a head-to-tail assembly, creating a composite active site that integrates the BB loop of one TIR domain with the DD loop of its neighbor—a configuration analogous to the *Sf*STING filament and other TIR-containing NADases (Fig. S5C) (44, 45). This structural arrangement highlights the necessity of straight filament formation for NADase activity, establishing it as a conserved activation mechanism across TIR-domain systems (44, 45). TIR domain assemblies are broadly divided into two structural classes: scaffold-type assemblies, which form parallel TIR domain columns (e.g., AbTir) (46), and enzymatic-type assemblies, characterized by antiparallel configurations (e.g., ROQ1 and RPP1) (47, 48). *El*STING belongs to the enzymatic class, assembling into open-ended antiparallel TIR domain strands—a structural signature conserved in proteins such as SARM1 (40, 44, 45). Although TIR-domain signaling universally relies on head-to-tail interactions between adjacent domains, *El*STING displays a distinct strand orientation within its antiparallel architecture. Strikingly, however, our data confirm that *El*STING activation strictly adheres to the canonical head-to-tail association rule. This mechanistic conservation transcends structural diversity across TIR-domain systems, unifying a core principle that governs their signaling outputs.

Another intriguing finding is that calcium ions play a crucial role in inhibiting the formation of the *El*STING fiber bundle and thereby suppressing the NADase activity of the TIR domain (Fig. 7). Previous studies have demonstrated that calcium ions are essential for various stages of the phage life cycle (49–53). During phage infection, unlimited replication of phage probably causes the depletion of calcium ions in the host, which may serve as a signal to be sensed by *El*STING. In the context of the CBASS, this depletion and the presence of 3′3′-c-di-GMP may act as a trigger to promote *El*STING activation. Therefore, calcium can be considered a second messenger that fine-tunes the strength of the host response.

The stepwise assembly of TIR-STING, as exemplified by *El*STING, may indicate a fine-tuned mechanism involving the regulation of activation of abortive infection. This modular activation process functions similarly to ancillary Cap proteins, which regulate CD-NTase activity in CBASS operons. Through fiber bundle formation to get activated, *El*STING ensures its NADase activity is tightly controlled, preventing unintended cellular damage, and such regulation is particularly crucial in scenarios where ancillary Cap proteins are absent. This stepwise assembly process may also serve as a safeguard mechanism against accidental activation of STING, which could otherwise lead to unwanted self-destructive activity in the absence of any genuine phage threat. The intricate interplay between structural changes and functional activation in *El*STING highlights a sophisticated regulatory strategy within CBASS, providing bacteria a balance between rapid response to infection and minimizing collateral damage. In a broader context, bacteria may employ sequential and progressive anti-phage strategies upon detecting infection. Early mechanisms, such as the CRISPR system, hold the frontline of defense while the prime backup strategies, including suicide pathways mediated by structures like spiral-shaped inactive STING are standing by. If the CRISPR system has successfully neutralized the phage, the abortive infection program would remain inactive. However, should CRISPR fail, unchecked phage replication will trigger signaling molecule fluctuations, such as 3′3′-c-di-GMP accumulation and calcium ion depletion to activate the abortive infection program. Such a control mechanism culminates in fiber bundle formation by *El*STING, providing bacteria a flexible and hierarchical response to phage elimination. These findings could also give a hint on the evolution of STING pathways in higher organisms, which may hypothetically link the bacterial CBASS systems to the innate immune responses in eukaryotes.

## MATERIALS AND METHODS

### Protein expression and purification

The *Epilithonimonas lactis* TIR-STING (WP_034976296.1) and *El*CdnE (WP_034976293.1) genes were codon-optimized, synthesized by Shanghai Logenbio Company, and cloned into the modified pMAL-c2x vector. Constructs for *El*STING and *El*CdnE were designed to produce an MBP-fusion protein with an HRV 3C protease cleavage site strategically placed between the MBP-His6 tag and the *El*STING protein. The expression and purification of wild-type *El*STING and its mutants follow the protocol described previously with modification (28). Briefly, expression of the *El*STING was carried out in *E. coli* BL21(DE3) pLysS cells grown in 2 × YT medium at 37°C. Induction was initiated with 0.2 mM IPTG, followed by overnight incubation at 16°C to optimize protein folding. After harvesting the bacterial cells, the pellets were resuspended in lysis buffer (25 mM Tris, 500 mM NaCl, pH 8.0) and disrupted. The resulting lysate was clarified via centrifugation, and the supernatant containing the target protein was applied to Ni-NTA resin (Cytiva) for affinity purification. Non-specific binding was minimized using a wash buffer (25 mM Tris, 500 mM NaCl, 25 mM imidazole, pH 8.0), and the MBP-fusion protein was eluted with elution buffer (25 mM Tris, 500 mM NaCl, 250 mM imidazole, pH 8.0). To remove the MBP-His6 tag, the eluate was incubated with HRV 3C protease overnight at 4°C. Cleaved MBP and residual uncleaved protein were removed by rebinding to Ni-NTA resin. The flow-through containing *El*STING was further purified by anion-exchange chromatography using a RESOURCE Q column (Cytiva). Proteins were eluted with a linear NaCl gradient (0–1 M) in 20 mM Tris (pH 8.0). A final polishing step was performed via size-exclusion chromatography (Superdex 200 Increase, Cytiva) in 20 mM Tris (pH 8.0), 150 mM NaCl, and 1 mM DTT. *El*CdnE purification followed the same protocol but omitted the ion-exchange step. SDS-PAGE with Coomassie blue staining was used to confirm the integrity and purity of the protein. The purified *El*STING and *El*CdnE proteins were concentrated to approximately 5 mg/mL, flash-frozen in liquid nitrogen, and stored at −80°C for subsequent use.

### Cryo-EM sample preparation and data acquisition

For the *El*STING/3′3′-c-di-GMP sample, C-flat R 2/1 holey carbon grids were first glow discharged for 15 seconds using a Pelco easiGlow glow discharge unit, and 3.5 µL sample was applied to the surface of the grid at a temperature of 8°C and humidity level of 80%. Grids were then blotted for 2.5 s before being plunge-frozen in liquid ethane using Vitrobot Mark IV. The cryo-specimens were loaded onto a Titan Krios transmission electron microscope (TEM; Thermo Fisher) operated at 300 kV for data collection. The microscope is equipped with a GIF-Quantum energy filter (Gatan), which was used with a slit width of 10 eV. Automatic data collection was performed using EPU software. Images were recorded with a Gatan K2 direct electron detector operating in super-resolution counting mode at a pixel size of 0.83 Å. The exposure was performed with a dose rate of 15 e^−^/pixel/s and an accumulative dose of ~50 e^−^/Å2 for each image which was fractionated into 36 movie frames. The final defocus ranges of the data sets were approximately 1.0–2.0 μm.

### Image processing and 3D reconstruction

For the *El*STING spiral-shaped filament, the dose-fractionated image stacks were subjected to beam-induced motion correction using MotionCor2 (54). Initial contrast transfer function (CTF) values for each micrograph were calculated with CTFFIND4 (55). Micrographs with an estimated resolution limit worse than 6 Å were discarded in the initial screening. A set of ~2,000 particles was manually picked and subjected to 2D classification to generate templates for auto-picking against the entire data set. The subsequent image processing and reconstruction were performed using cryoSPARC (56). 1,120,445 particles were picked from 10,585 micrographs. Then the picked particles were extracted and subjected to two rounds of reference-free 2D classification in cryoSPARC, which yielded 523,122 particle projections. This subset was subjected to one round of Hetero refinement. The predominant class containing a subset of 264,700 best particles shows the clear features of secondary structural elements. These particles were subjected to a Non-uniform refinement with C2 symmetry, which yielded a reconstruction at 2.88 Å resolution (Fig. S1).

The *El*STING fiber bundle data were processed similarly to the above *El*STING single filament data. The detailed image processing and reconstruction are shown in Fig. S2. Local resolution estimate was performed with cryoSPARC.

### Model building and refinement

The initial model of *El*STING was generated using AlphaFold3 and subsequently docked into electron microscopy (EM) density maps. For the spiral-shaped *El*STING filament, the predicted model fit seamlessly into the density map. After several rounds of refinement, the quality of the *El*STING protomer model was significantly enhanced.

The spiral-shaped *El*STING octamer model was constructed by docking the refined protomer model into the remaining regions of the density map. For the *El*STING fiber bundle structure, the predicted model was initially docked into the EM map, and discrepancies—particularly in the CBDα4 region—were manually adjusted. The octamer fiber bundle was then assembled by docking the refined protomer model into the corresponding regions of the map. Refinement of these models against the EM map was performed using Phenix.real_space_refine (57), and structural quality was assessed using MolProbity (Table S1) (58). Structural analyses and figure rendering were conducted using PyMOL (59), Chimera (60), and ChimeraX (61).

### Cyclic dinucleotide synthesis analysis

*El*CdnE enzymatic activity was assessed by monitoring cyclic dinucleotide production via HPLC. The reactions were conducted in a total volume of 300 µL containing 20 mM HEPES, 100 mM KCl, 50 µM of each NTP (ATP, GTP, CTP, UTP; 200 µM total), 5 µM purified *El*CdnE, and 10 mM MgCl_2_. After incubation at 37°C for 3 hours, the reactions were terminated by heating at 100°C for 10 minutes. The mixtures were centrifuged at 14,000 rpm for 10 minutes and filtered through 0.45 µm membranes to remove particulates. The prepared samples were injected onto a Sapphire C18 column (4.6 × 250 mm, 100 Å, 5 µm) connected to a Waters 2695 system. Product separation and analysis were carried out via HPLC with absorbance detection at 254 nm. An isocratic elution strategy was employed, using an elution buffer of 20 mM KH_2_PO4 (pH 5.4) with 6% ethanol, at a flow rate of 0.7 mL/min and a column temperature of 20°C.

### Cell toxicity assay

Wild-type and mutant *El*STING constructs were cloned into the pET28a vector (Merck) to encode C-terminal His6-tagged proteins and transformed into BL21(DE3) pLysS cells. Transformed colonies were inoculated into LB medium and grown overnight at 37°C with shaking (220 rpm) to generate starter cultures. For cytotoxicity assays, overnight cultures were diluted 1:100 in fresh LB medium to an initial OD_600_ of ~0.01, and protein expression was immediately induced with 0.1 mM IPTG. Bacterial growth was monitored hourly for 8 hours by measuring OD_600_ using a spectrophotometer (Eppendorf). Growth curves were generated by plotting OD_600_ values against time to assess viability differences between wild-type and mutant constructs.

### Negative-stain TEM

*El*STING protein and its variants were incubated with specified ligands (protein:ligand molar ratio = 1:1, 1 mM EDTA, or 1 mM CaCl₂) overnight at 4°C. Samples were diluted to a final concentration of 50 µg/mL in TEM sample buffer (20 mM Tris, 150 mM NaCl, pH 8.0). Carbon-coated electron microscope grids were glow-discharged for 40 s immediately prior to use to enhance surface hydrophilicity. A parafilm strip, secured to the bench surface with water, was prepared to sequentially deposit 6 µL droplets of the protein sample, sample buffer, and 1% (wt/vol) uranyl acetate solution. Glow-discharged grids (carbon side facing down) were floated on the protein sample droplet for 1 min, followed by brief blotting of excess liquid using filter paper. The grids were then transferred to the sample buffer droplet for 5 s and subsequently to the uranyl acetate stain for 1 min, with blotting performed after each step to remove residual solution. Stained grids were air-dried at ambient conditions, stored in an EM grid box, and imaged using an FEI Tecnai Spirit 100 kV transmission electron microscope operated at 98,000× magnification.

### Phage amplification and storage

*Escherichia* phage T2 (DSM 16352) was obtained from the Deutsche Sammlung von Mikroorganismen und Zellkulturen GmbH (DSMZ). The host strain, *Escherichia coli* BL21(DE3), was cultured in 20 mL of LB medium under shaking conditions (200 rpm) until the culture reached an optical density at 600 nm (OD_600_) of approximately 1. Subsequently, 100 µL of phage stock solution was added to the culture, which was then incubated overnight with continued shaking at 200 rpm to allow for phage propagation and host lysis. After lysis was observed, a few drops of chloroform were added to the culture to ensure complete lysis, and the mixture was gently mixed. The lysate was transferred to centrifuge tubes and centrifuged at 5,000 rpm for 10 minutes to separate bacterial cells and debris. The resulting supernatant was carefully filtered through a 0.45 µm nitrocellulose membrane to obtain a clear and stable phage lysate.

The titer of the phage lysate was quantified using a double-layer agar plaque assay. The prepared phage stock solution was stored at 4°C for subsequent use.

### Phage infection assay

*E. coli* naturally expresses endogenous 3′3′-c-di-GMP, and the recognition of these molecules by overexpressed *El*STING in the cell induces toxicity, thereby preventing phage infection. This mechanism forms the basis of this assay. The plasmids pET28a-*El*STING and its mutants were transformed into the BL21(DE3) pLysS expression strain. The transformed cells were inoculated into 5 mL of LB medium and incubated overnight at 37°C with shaking. The overnight culture was then diluted with fresh LB medium to an optical density at 600 nm (OD_600_) of approximately 0.1. The cells were initially infected with T2 phage at a multiplicity of infection (MOI) of 0.01 for 30 minutes. Subsequently, 0.5 mM IPTG was added to induce the overexpression of the target protein. After 6–8 hours of incubation, the growth of the phage titer was evaluated using a double-layer agar plaque assay. Data analysis was performed using GraphPad Prism 8.0.2 software. Results are presented as the mean of three independent biological replicates, with error bars representing the standard deviation.

### NAD^+^ cleavage assay

HPLC was employed to measure the NAD^+^ cleavage activity of *El*STING. The reactions were conducted in a total volume of 300 µL containing 20 mM HEPES, 100 mM KCl, 500 µM β-nicotinamide adenine dinucleotide (NAD+; Sigma), 2 µM test proteins, and 20 µM 3′3′-c-di-GMP. To investigate the influence of calcium ions on enzymatic activity, 1 mM EDTA or 2 mM CaCl_2_ was added to the reaction buffer. After incubation at 37°C for 3 hours, the reactions were terminated by adding 1 M HCl. The mixtures were centrifuged at 14,000 rpm for 10 minutes and filtered through 0.45 µm membranes to remove particulate matter. The prepared samples were injected onto a Sapphire C18 column (4.6 × 250 mm, 100 Å, 5 µm) connected to an Agilent 1260 I system. Product separation and analysis were carried out via HPLC with absorbance detection at 260 nm. An isocratic elution strategy was employed, using an elution buffer of 20 mM KH_2_PO_4_ (pH 5.4) with 6% ethanol, at a flow rate of 0.7 mL/min and a column temperature of 20°C. Product peaks were integrated for each sample, and relative NAD^+^ cleavage activity was calculated. Results, derived from three independent biological replicates, are presented with error bars indicating the standard deviation.

### Gel filtration assay

*El*STING protein was incubated with 3′3′-cyclic dinucleotides (CDNs)—including 3′3′-c-di-GMP, 3′3′-c-di-AMP, 3′3′-cGAMP, and 2′3′-cGAMP—at a 1:1 molar ratio (protein:ligand) overnight at 4°C. For 3′3′-c-di-GMP, additional experiments were conducted in the presence of 1 mM EDTA or CaCl₂ to assess ion-dependent effects. Samples were loaded onto a Superose 6 Increase 10/300 Gl column (Cytiva) pre-equilibrated with gel filtration buffer (20 mM Tris, 150 mM NaCl, 1 mM DTT, pH 8.0). Chromatography was performed at a constant flow rate of 0.5 mL/min, and elution profiles were monitored via UV absorbance at 280 nm.

## SUPPLEMENTAL MATERIAL

Supplemental material mbio.00388-25-s0001.docx Fig. S1-S6 and Table S1.

ASM does not own the copyrights to Supplemental Material that may be linked to, or accessed through, an article. The authors have granted ASM a non-exclusive, world-wide license to publish the Supplemental Material files. Please contact the corresponding author directly for reuse.
