# Supplementary material for: Structural insights into distinct filamentation states reveal a regulatory mechanism for bacterial STING activation
Source: mBio. 2025 Aug 14;16(9):e00388-25. doi: 10.1128/mbio.00388-25 (PMC12421889; doi:10.1128/mbio.00388-25)
Supplement: Supplemental material — Fig. S1-S6 and Table S1. [file mbio.00388-25-s0001.docx]

**Supplementary Information**

**Structural insights into distinct filamentation states reveal a regulatory mechanism for bacterial STING activation**

**Yang et al**

**
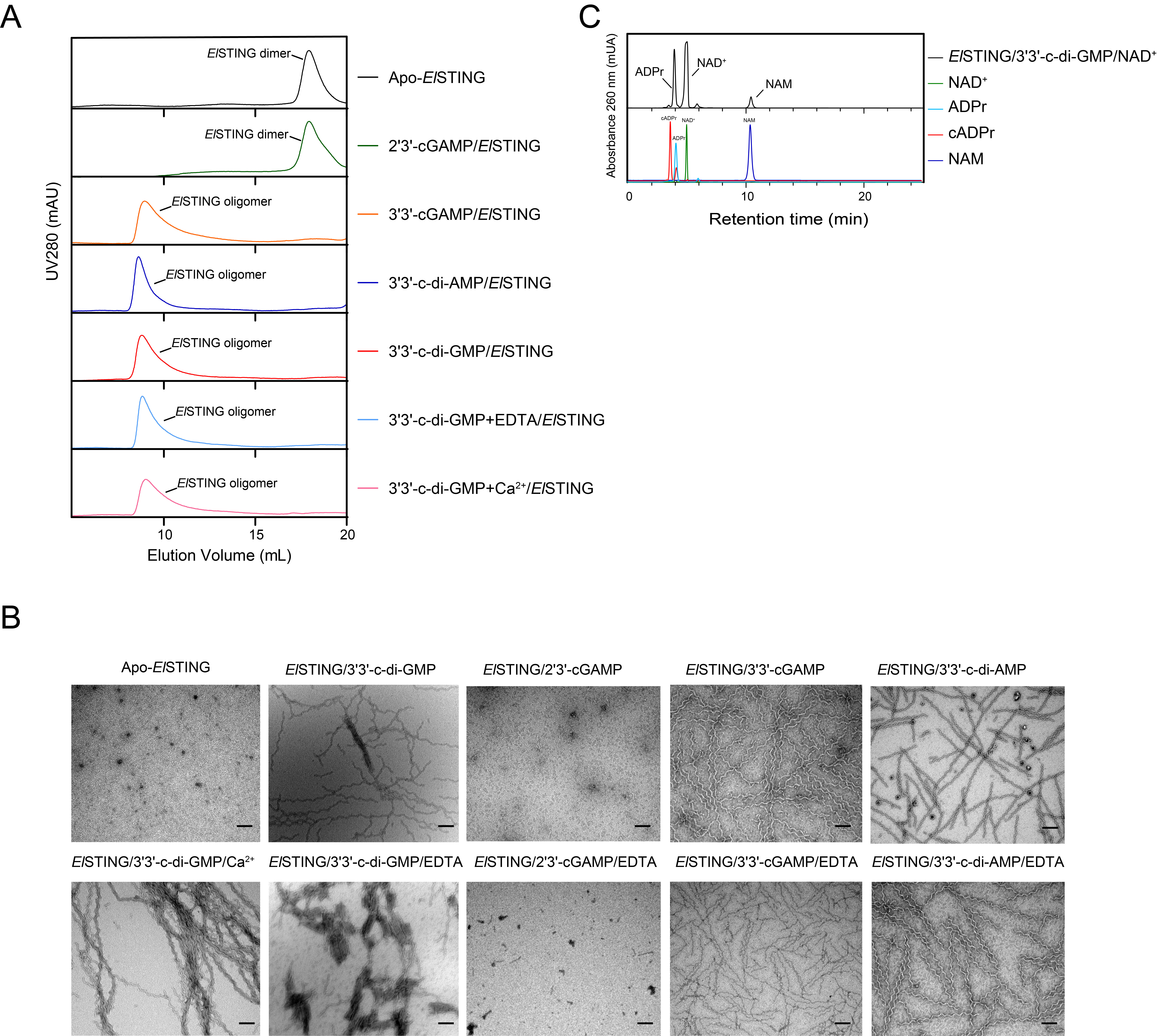
**

**Supplementary Figure 1. Characterization of *El*STING oligomerization in the presence of various cyclic dinucleotides.**

(A) Size exclusion chromatography (SEC) (Superose 6 Increase 10/300 GL) analysis of *El*STING under different conditions. UV absorbance at 280 nm (mAU) is plotted against elution volume (mL). *El*STING forms dimers in the absence of ligands (Apo-*El*STING) and oligomerizes upon binding to 3'3'-c-di-GMP, 3'3'-cGAMP and 3'3'-c-di-AMP, excepting 2'3'-cGAMP.

(B) Negative-stain EM micrographs demonstrating CDN-induced *El*STING oligomer formation. Scale bars = 100 nm.

(C) Identification of the products of NAD+ cleaved by *El*STING in the presence of 3'3'-c-di-GMP using HPLC.

**
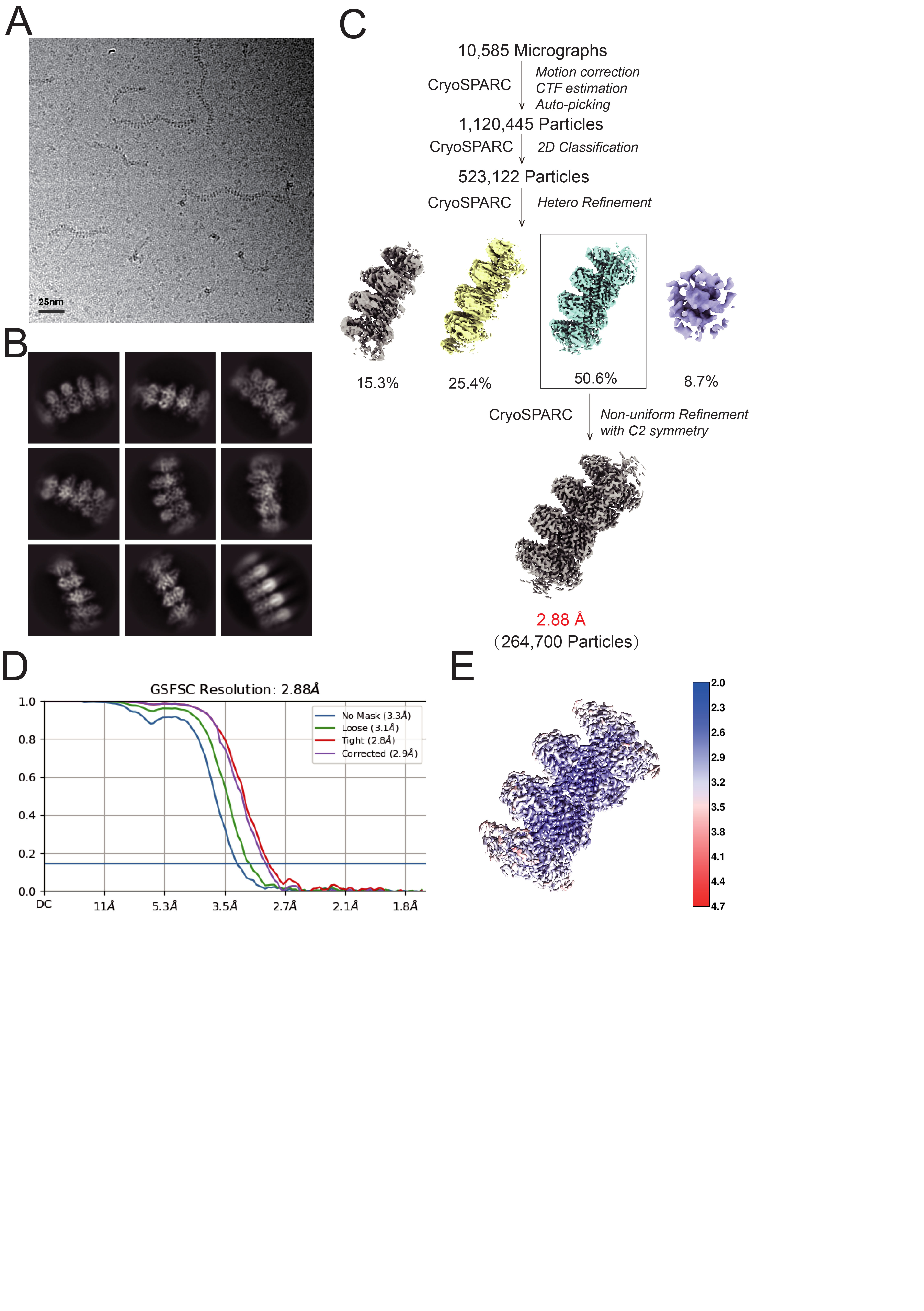
**

**Supplementary Figure 2. Cryo-EM analysis of the spiral-shaped *El*STING/3'3'-c-di-GMP structure.**

(A) Representative cryo-EM micrograph of the spiral-shaped *El*STING polymer. Scale bar: 25 nm.

(B) 2D class averages of the spiral-shaped *El*STING polymer.

(C) Schematic workflow of cryo-EM image processing and reconstruction.

(D) Gold-standard Fourier shell correlation (GSFSC) curve for the reconstruction, showing resolution assessment.

(E) Local resolution distribution of the density map for the spiral-shaped *El*STING polymer.

**
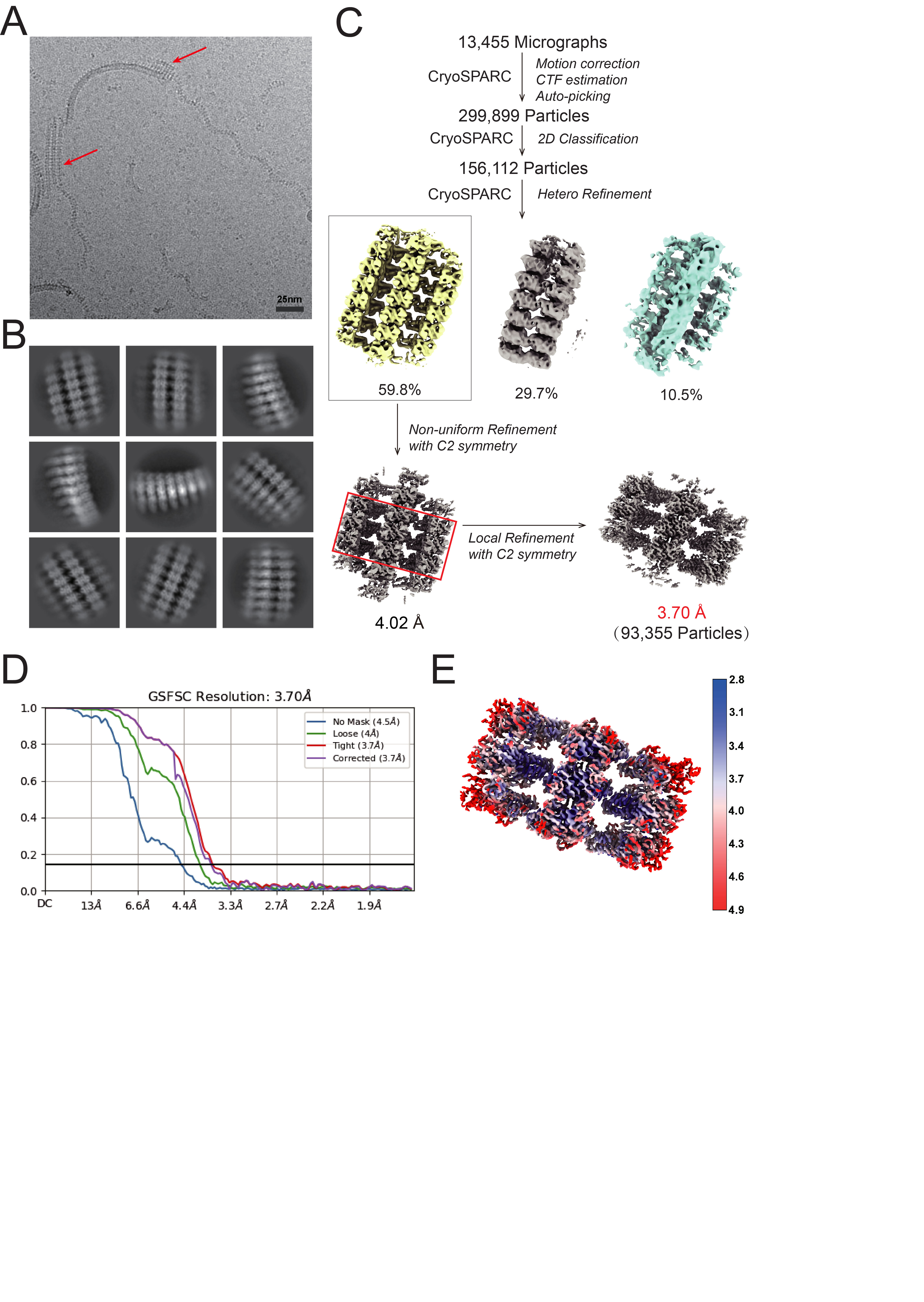
**

**Supplementary Figure 3. Cryo-EM analysis of the *El*STING fiber bundle.**

(A) Representative cryo-EM micrograph of the *El*STING fiber bundle (indicated by red arrows). Scale bar: 25 nm.

(B) 2D class averages of the *El*STING fiber bundle.

(C) Schematic workflow of cryo-EM image processing and reconstruction.

(D) Gold-standard Fourier shell correlation (GSFSC) curve for the reconstruction, providing resolution assessment.

(E) Local resolution distribution of the density map for the *El*STING fiber bundle.


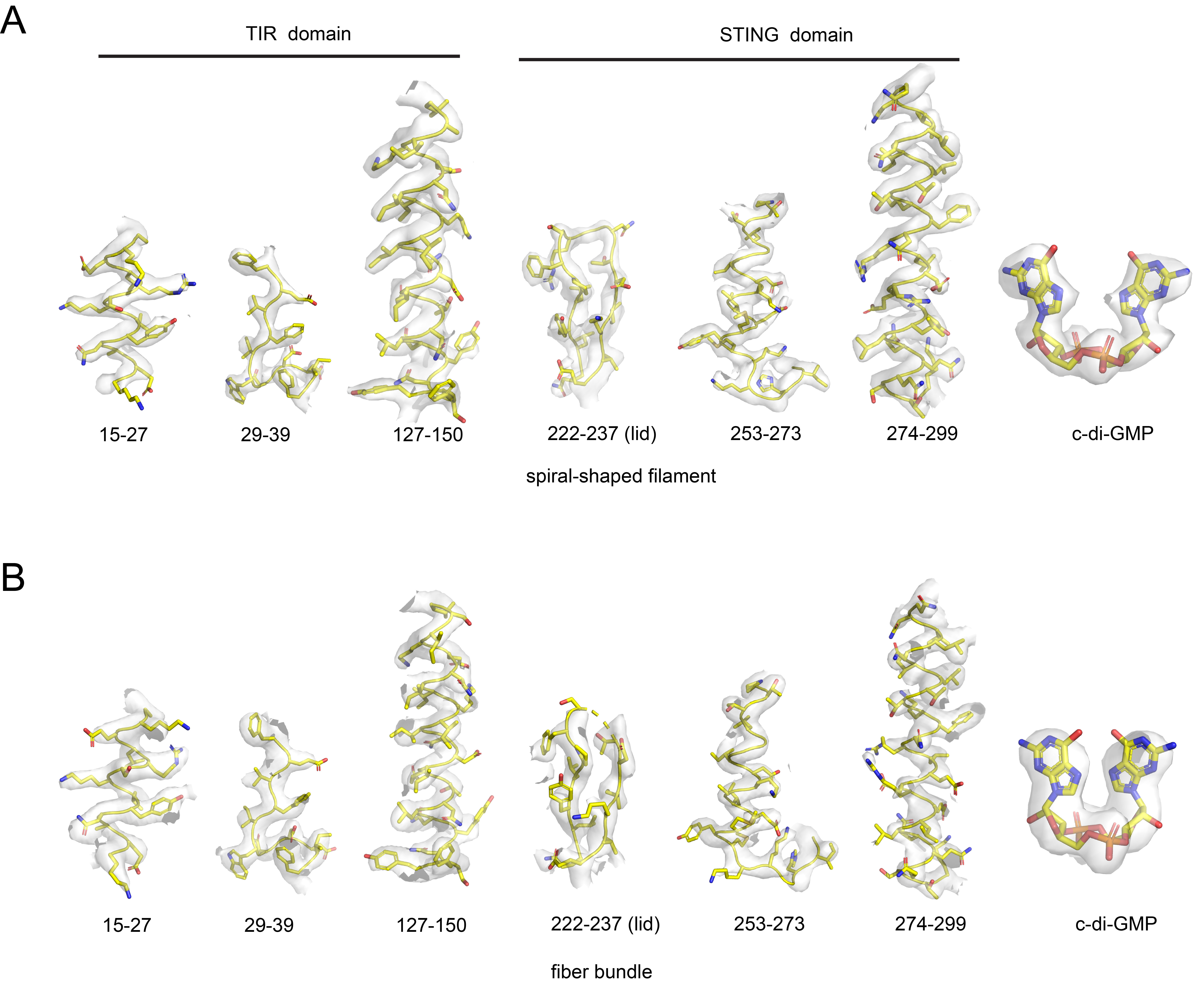


**Supplementary Figure 4. Examples of map-to-model fit quality for select regions of the *El*STING/3'3'-c-di-GMP complex (contoured at 6σ).**

(A) Expanded views of density maps highlighting various regions of the *El*STING/3'3'-c-di-GMP spiral-shaped structure, showing model-to-map fit quality.

(B) Expanded views of density maps for select regions of the *El*STING/3'3'-c-di-GMP fiber bundle structure, illustrating model-to-map fit quality.


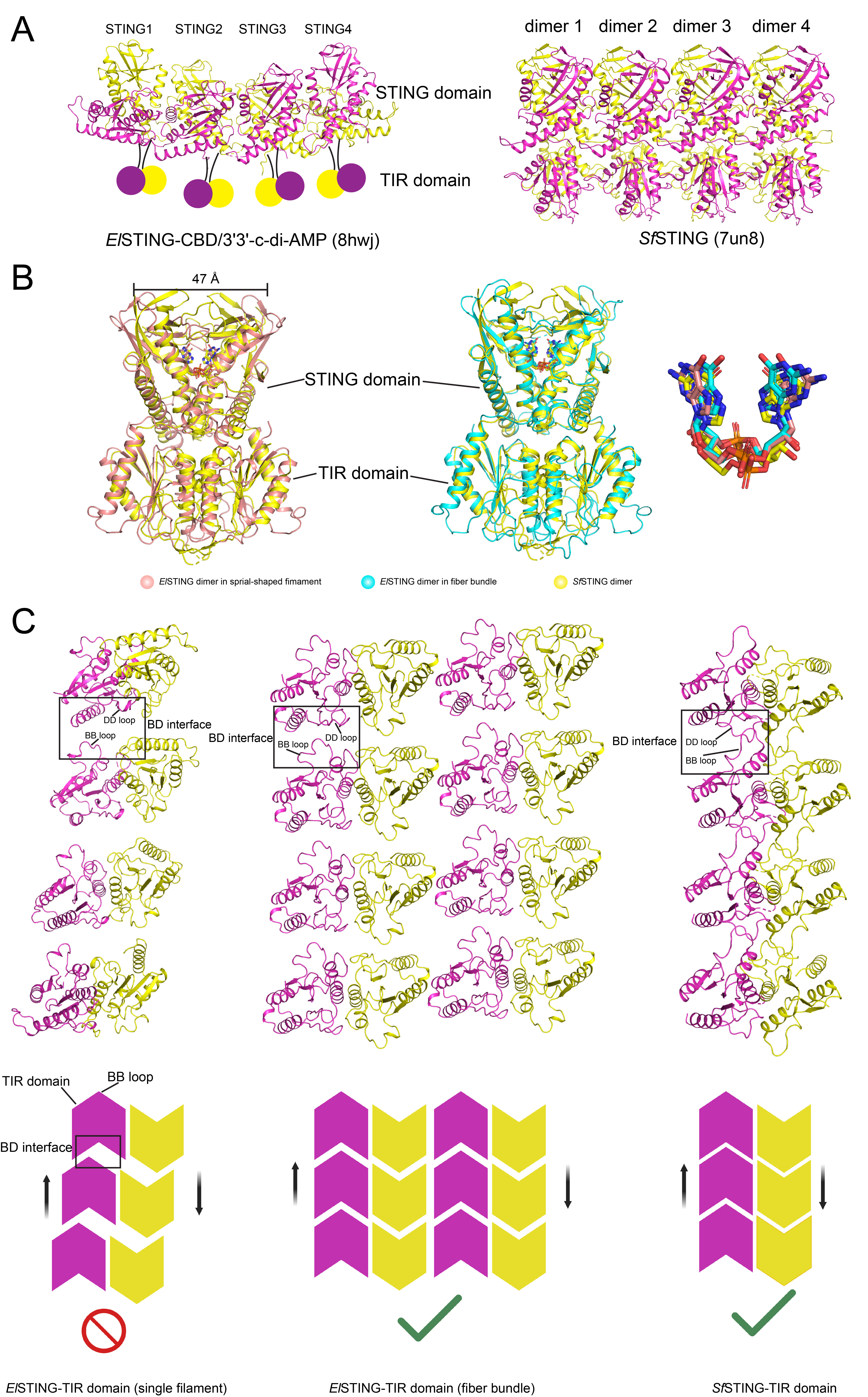


**Supplementary Figure 5. Structural and functional comparison of *El*STING and *Sf*STING oligomerization states.**

(A) Cartoon representations of *El*STING/3'3'-c-di-AMP oligomers (left, inactive spiral filament; PDB: 8HWI) and *Sf*STING filaments (right, active state; PDB: 7UN8). The *Sf*STING filament adopts a linear, active conformation, while *El*STING forms a calcium-independent spiral filament (inactive) in crystal lattice. (B) Superimposition of the *Sf*STING (yellow) dimer with *El*STING dimer from the spiral-shaped filament (salmon) and fiber bundle (cyan) respectively, shown as cartoons. The dimensions (47 Å) between the tips of the *Sf*STING protomers is labeled. Overlay of c-di-GMP binding conformations across the three structures (right panel). (C) Distinct TIR domain assembly mechanisms. Top panel: In *El*STING single filaments (left, inactive), the BB loop is far away from DD loop from adjacent TIR in BD interface and cannot form the active site. In *El*STING fiber bundle (middle, active), the BB loop could interact with DD loop from adjacent TIR in BD interface and form active site, probably induced by substrate binding. In *Sf*STING filaments (right, active), BB loop interacting with DD loop from adjacent TIR in BD interface forms active site. Bottom panel: Schematics highlight assembly modes. Checkmarks denote active states; Prohibitive signs indicate inactive conformations. *El*STING activation necessitates fiber bundle formation (head-to-tail TIR alignment), contrasting with *Sf*STING’s single-filament activation.


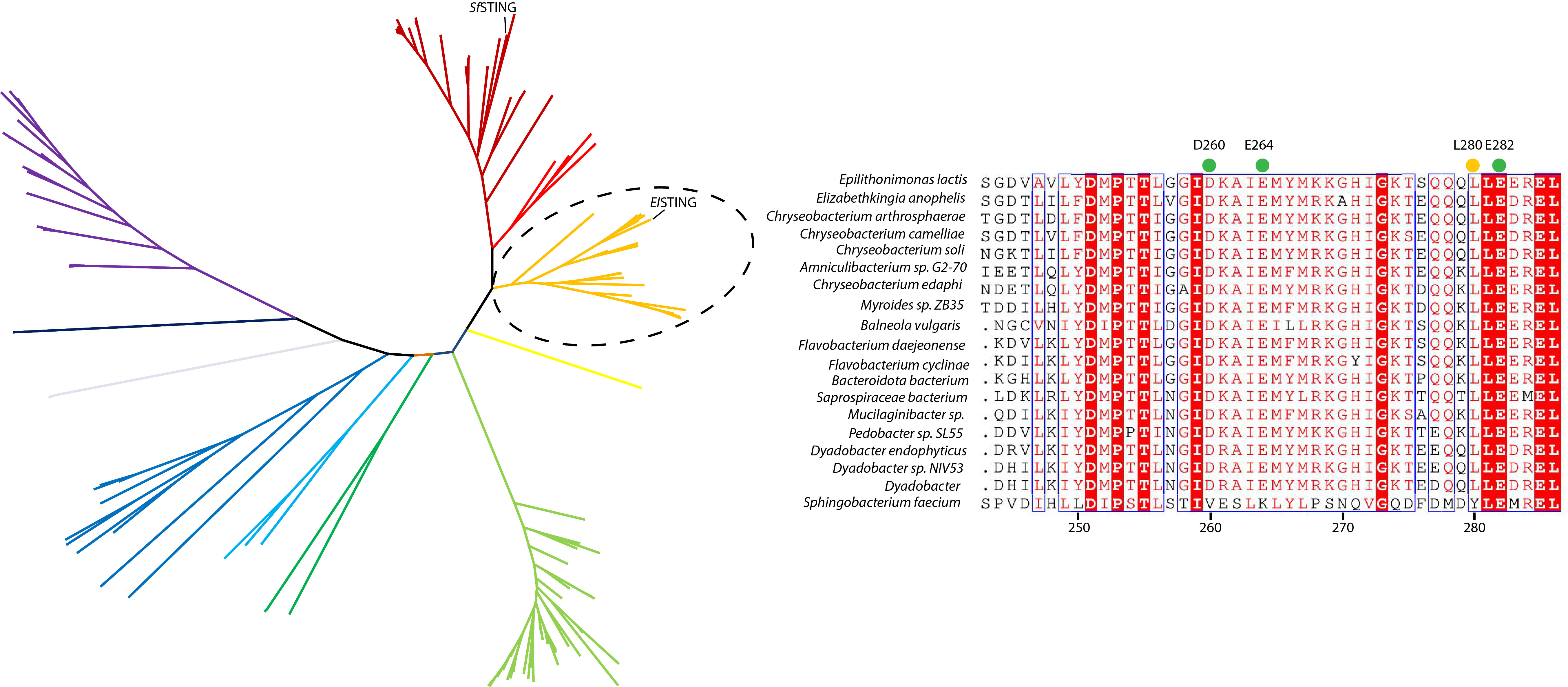


**Supplementary Figure 6. Phylogenetic and sequence analysis of TIR-STING proteins.** Left panel: Phylogenetic tree constructed from 127 TIR-STING-related sequences with 30–75% sequence identity. Different clades are color-coded, with *El*STING and *Sf*STING distinctly labeled. *El*STING clusters within a specific clade (outlined with a dashed line), indicating a close relationship among these sequences. Right panel: Multiple sequence alignment comparing *Sf*STING with the *El*STING-containing clade. Conserved residues critical for calcium binding (highlighted in green) and TIR domain inhibition (highlighted in orange) are shown, emphasizing their functional conservation. Residue numbers correspond to *El*STING.

Supplementary table 1

**Cryo-EM data processing and refinement statistics.**

|  | Spiral-shaped *El*STING/3'3'-c-di-GMP oligomer (PDB ID: 9LMQ) | | *El*STING/3'3'-c-di-GMP fiber bundle structure (PDB ID: 9LMR) |
| --- | --- | --- | --- |
| **Data collection and processing** | | | |
| Magnification | 105k | 105k | |
| Voltage (kV) | 300 | 300 | |
| Electron exposure (e^–^/Å^2^) | 50 | 50 | |
| Defocus range (μm) | -1.0 to -2.0 | -1.0 to -2.0 | |
| Pixel size (Å) | 0.83 | 0.83 | |
| Symmetry imposed | C2 | C2 | |
| Final particle images (no.) | 264,700 | 93,355 | |
| Map resolution (Å)  FSC threshold | 2.88  0.143 | 3.70  0.143 | |
| Map resolution range (Å) | 2.0-4.5 | 2.8-5.5 | |
|  |  |  | |
| **Refinement** |  |  | |
| Initial model used (PDB code) | Alphafold 3 predicted model | 9LMQ | |
| Model resolution (Å) | 2.88 | 3.70 | |
| Map sharpening *B* factor (Å^2^) | -133.4 | -125.9 | |
| Model composition  Non-hydrogen atoms  Protein residues  Ligands | 20205  2480  CA: 4 C2E: 4 | 19952  2450  C2E: 4 | |
| *B* factors (Å^2^)  Protein  Ligand | 43.41  39.41 | 49.75  36.93 | |
| R.m.s. deviations  Bond lengths (Å)  Bond angles (°) | 0.002  0.478 | 0.004  0.684 | |
| **Validation**  MolProbity score  Clashscore  Poor rotamers (%) | 1.63  8.14  0.00 | 2.23  15.77  0.00 | |
| Ramachandran plot  Favored (%)  Allowed (%)  Disallowed (%) | 96.86  3.14  0.00 | 90.55  9.28  0.17 | |
